# Endosomal signalling via exosome surface TGFβ-1

**DOI:** 10.1080/20013078.2019.1650458

**Published:** 2019-09-20

**Authors:** Ganesh Vilas Shelke, Yanan Yin, Su Chul Jang, Cecilia Lässer, Stefan Wennmalm, Hans Jürgen Hoffmann, Li Li, Yong Song Gho, Jonas Andreas Nilsson, Jan Lötvall

**Affiliations:** a Krefting Research Centre, Institute of Medicine, the Sahlgrenska Academy, University of Gothenburg, Gothenburg, Sweden; b Department of Surgery, Institute of Clinical Sciences, the Sahlgrenska Academy, University of Gothenburg, Gothenburg, Sweden; c Department of Biochemistry and Molecular Cell Biology, Shanghai Jiao Tong University, School of Medicine, Shanghai, China; d Royal Institute of Technology-KTH, Department of Applied Physics, Experimental Biomolecular Physics Group, SciLife Laboratory, Solna, Sweden; e Department of Clinical Medicine, Aarhus University, Aarhus, Denmark; f Department of respiratory and Allergy, Aarhus University Hospital, Aarhus, Denmark; g Department of Laboratory Medicine, Shanghai First People’s Hospital, Shanghai JiaoTong University, Shanghai, China; h Department of Life Sciences, Pohang University of Science and Technology, Pohang, Republic of Korea

**Keywords:** Mast cells, extracellular vesicles, exosomes, mesenchymal stem cells, tumour growth factor beta-1, cellular localization, endosomal signalling, proteoglycan

## Abstract

Extracellular vesicles such as exosomes convey biological messages between cells, either by surface-to-surface interaction or by shuttling of bioactive molecules to a recipient cell’s cytoplasm. Here we show that exosomes released by mast cells harbour both active and latent transforming growth factor β-1 (TGFβ-1) on their surfaces. The latent form of TGFβ-1 is associated with the exosomes via heparinase-II and pH-sensitive elements. These vesicles traffic to the endocytic compartment of recipient human mesenchymal stem cells (MSCs) within 60 min of exposure. Further, the exosomes-associated TGFβ-1 is retained within the endosomal compartments at the time of signalling, which results in prolonged cellular signalling compared to free-TGFβ-1. These exosomes induce a migratory phenotype in primary MSCs involving SMAD-dependent pathways. Our results show that mast cell-derived exosomes are decorated with latent TGFβ-1 and are retained in recipient MSC endosomes, influencing recipient cell migratory phenotype. We conclude that exosomes can convey signalling within endosomes by delivering bioactive surface ligands to this intracellular compartment.

## Introduction

Cell-to-cell communication can occur via secreted soluble mediators such as cytokines, bioactive proteins and nucleotides [1–4]. After their release from the cell, they act in an autocrine or paracrine manner to modulate cellular function. To achieve longer half-life, secreted mediators can either exist in an inactive conformation by binding to pro-protein or interact with extracellular matrix proteins, which requires activation before a biological signal can be conveyed [5–7]. Activation can happen either by matrix degradation or by proteolytic cleavage of matrix or pro-protein to release bioactive molecules like transforming growth factor beta-1 (TGFβ-1) [5,8].

In circulation, TGFβ-1 exists in an active and inactive form in a ~ 1:65 ratio [9]. Inactive TGFβ-1 exists as a dimer of pro-proteins consisting of latent binding protein (LAP) and active-TGFβ-1 subunit. The role of active-TGFβ-1 in activating the SMAD/non-SMAD signalling pathway has been extensively studied [10–12]. However, the exact mechanisms of inactive-TGFβ-1 activation are not well studied.

Previous studies have reported the association of TGFβ-1 with secretory structures called extracellular vesicles (EVs) [13,14]. EVs, including exosomes and microvesicles, are nano-to-micrometre sized, lipid bilayer-enclosed membrane structures that carry an array of bioactive molecules, including proteins, nucleic acids, and lipids. In this study the term “exosomes” is used for EVs, as the isolation procedures we use are known to enrich for small EV. Extensive proteomics of exosomes has highlighted the presence of TGFβ-1 in those preparations [15–18]. Therefore, it would be important to determine how the TGFβ-1 is associated with the exosomes, including its topology. TGFβ-1 secreted from immune cells regulates many processes, including migration and immunomodulation of recipient cells such as human mesenchymal stem cells (MSCs). MSCs can regulate immune responses, which has made them attractive therapeutic candidates in several inflammatory diseases [19–24].

We hypothesized that highly bioactive surface molecules on exosomes can convey rapid signalling in recipient cells. To investigate this, we first performed membrane proteomics of exosomes released by mast cells, and specifically looked for the presence of TGFβ-1. We then confirmed the association of the form of TGFβ-1 to the exosome membranes and its coexistence with characteristic exosome-associated proteins at single-vesicle resolution. Lastly, the mechanism by which TGFβ-1 is associated with the membrane was determined. Exosomes can rapidly traffic to the endosomal compartment; thus, we also tested whether TGFβ-1 can induce recipient cell signalling in the intercellular membrane compartment.

## Methods

### Cell culture

Human bone marrow-derived mesenchymal stem cells (MSCs) were obtained at passage 1 from the MSC distribution at the Institute of Regenerative Medicine at Scott and White, USA. The MSCs were cultured in Minimum Essential Medium α-GlutaMAX™ Supplement (Life Technologies, Thermo Fisher Scientific, Waltham, MA, USA) supplemented with 15% fetal bovine serum (FBS; Sigma Aldrich, St. Louis, MO, USA). The culture media was changed to EV-depleted FBS-containing medium 24 h prior to experiments. The MSCs were used within 3–4 passages in all experiments, with a seeding density of 3000 cells/cm^2^. The human mast cells, HMC-1 (J, Butterfield, Mayo Clinic, Rochester, MN, USA), were grown in Iscove’s modified Dulbecco’s medium (IMDM, HyClone Laboratories, Logan, UT, USA) supplemented with 10% EV-depleted FBS, 2 mM L-glutamine (HyClone Laboratories), and 1.2 mM α-thioglycerol (Sigma Aldrich). HEK293T cells (ATCC, Manassas, VA, USA) were grown in RPMI-1640 medium (HyClone Laboratories) supplemented with 10% FBS. All cultures were supplemented with 100 units/ml penicillin and 100 µg/ml streptomycin (HyClone Laboratories). Here, the supplement FBS was depleted of EVs by ultracentrifugation for 18 h at 120,000 × *g* (Type 45 Ti rotor, Beckman Coulter) as described earlier [57]. All cells were cultured at 37°C in a 5% CO_2_ humidified atmosphere. For the purification and culture of progenitor mast cells, we used PBMC from healthy human donors. Briefly, mononuclear cells were purified from PBMC, and the CD133^+^ cells were isolated by MACS (Miltenyi Biotech, Germany). CD133^+^ cells were cultured in a serum-free medium (StemSpan, StemCell technology, Vancouver Canada) supplemented with SCF and IL-6. IL-3 was added for the first 2 weeks, and IL-4 for the last 2 weeks. Cells were then maintained for 6–7 weeks before the conditioned medium was harvested for exosomes isolation [58].

## Isolation of exosomes

### Using ultracentrifugation pelleting

Exosomes were isolated from conditioned cell medium by differential centrifugation and a filtration step, as previously described. Briefly, 3–4-day culture medium was centrifuged at 300 × *g* for 10 min to remove cells. The supernatant was further centrifuged at 16,500 × *g* for 20 min. Subsequently, the supernatant was centrifuged at 120,000 × *g* for 3 h (Type 45 Ti rotor, Beckman Coulter). The samples were dissolved in PBS, and the protein concentration was measured by a BCA Protein assay kit (Pierce™, Thermo Fisher Scientific, Waltham, MA, USA). We used this type of exosome preparation in all studies unless indicated.

### Using density cushion

In some experiments (Figure 6 and Supplementary Figure 4), exosomes were collected on 10–30% iodixanol interphase cushions instead of direct pelleting (Supplementary Figure 3a). After collecting the exosomes from the interphase, they were bottom loaded onto an iodixanol flotation gradient (0, 20, 22, 24, 26, 28, 30, 50, 60%) followed by subsequent flotation by centrifuging at 182,300 × *g* for 16 h using an SW40-Ti swinging bucket rotor. Purified exosomes were collected from fractions between layers 20% and 22% after centrifugation.

### Reversed cell migration and invasion assay

The migration capacity and invasiveness of MSCs were evaluated using a 48-well Boyden chamber (Neuroprobe, Gaithersburg, MD, USA). In some experiments, MSCs were pre-incubated with mast cell-derived exosomes for 48 h before seeding and referred to as exosome-treated MSCs. Five thousand cells/well were seeded to the bottom compartment and were separated from the upper chamber by a polycarbonate membrane with 8 µm pores. The membrane was pre-coated with 0.1% gelatin or 200 μg/ml ECM Gel from Engelbreth-Holm-Swarm murine sarcoma (Sigma-Aldrich). After being seeded, cells were allowed to adhere onto the membrane by inverting the chamber assembly upside down for 3.5 h. Later the chamber was placed in the correct orientation and FBS was added in the upper compartment. After incubation for 12 h at 37°C, the membrane was removed and cells on the migrated sides were fixed in methanol (10 min) and stained with Giemsa (Histolab, Västra Frölunda, Sweden) for 1 h. Cells from the non-migrated side were wiped off before imaging. Three fields at 40× magnifications were imaged. For the migratory inhibition experiments, MSCs were incubated with 100 nM of LY2157299 (Selleckchem, Munich, Germany), which is an inhibitor of TGFβ type-1 receptor. Each analysis was performed in triplicate.

### Scratch assay

Human MSCs were grown to 70–80% confluence in 6-well plates, and the monolayer cells were scratched with a 1 ml pipette tip across the centre of the wells. After the cells had been washed with PBS, MEM plain medium with or without exosomes (100 μg/ml) was incubated with MSCs. Migratory cells from the scratched boundary were imaged after various time points.

### Gelatin zymography

The supernatant from MSCs, cultured with or without mast cell-derived exosomes, was collected at 24 and 48 h and electrophoresed onto zymogram precast gels containing 10% gelatin (BioRad Laboratories, Hercules, CA, USA) with 5× non-reducing loading buffer (Sigma Aldrich). Gels were re-natured with 2.5% Triton X-100 (Sigma Aldrich) for 1 h at room temperature and then incubated in development solution (50 mM Tris (pH 7.4), 5 mM CaCl_2_, 200 mM NaCl) at 37°C overnight. Gels were stained (coomassie brilliant blue) and destained (30% methanol and 10% acetic acid) until the clear bands appeared. Finally, the gel was incubated with stop solution (2% acetic acid). Band intensity was quantified using ImageJ software.

### Sample digestion and nanoLC-MS analysis to identify membrane proteins on exosomes

Proteomic analyses were performed at The Proteomics Core Facility at the Sahlgrenska Academy, University of Gothenburg. The samples in approximately 100 μl of PBS were lysed by the addition of sodium dodecyl sulfate (SDS) to a final concentration of 2% SDS and 50 mM triethylammonium bicarbonate (TEAB). Total protein concentration was determined with the Pierce™ BCA Protein Assay (Thermo Scientific). Aliquots containing 50 μg of each sample were digested with trypsin using the filter-aided sample preparation (FASP) method [59]. Briefly, protein samples were reduced with 100 mM dithiothreitol at 60°C for 30 min, transferred to 30 kDa MWCO Pall Nanosep centrifugal filters (Sigma-Aldrich), washed with 8M urea solution repeatedly, and alkylated by addition of methyl methanethiosulfonate to a final concentration of 10 mM. Digestion was performed in 50 mM TEAB, 1% sodium deoxycholate (SDC) overnight at 37°C after addition of trypsin (Pierce Trypsin Protease, MS Grade, Thermo Fisher Scientific) in a ratio of 1:100 relative to the amount of protein. An additional portion of trypsin was added and incubated for another 2 hours followed by the collection of the peptides by centrifugation. Samples were acidified to pH 2 by addition of TFA to precipitate SDC.

Samples were desalted using PepClean C18 spin columns (Thermo Fisher Scientific) according to the manufacturer’s guidelines prior to analysis on a Q Exactive mass spectrometer (Thermo Fisher Scientific) interfaced with Easy nLC 1000 liquid chromatography system. Peptides were separated using an in-house constructed C18 analytical column (200 × 0.075 mm I.D., 3 μm, Dr. Maisch, Germany) using a gradient from 5% to 25% acetonitrile in 0.1% formic acid for 75 min and finally from 25% to 80% acetonitrile in 0.1% formic acid for 5 min at a flow of 200 nL/min. Precursor ion mass spectra were recorded in positive ion mode at a resolution of 70,000 and a mass range of *m/z* 400 to 1600. The 10 most intense precursor ions were fragmented using HCD at a collision energy of 27, and MS/MS spectra were recorded in a scan range of *m/z* 200 to 2000 and a resolution of 35,000. Charge states 2 to 6 were selected for fragmentation, and dynamic exclusion was set to 30 s. Samples were re-analyzed with exclusion lists of *m/z* values of the identified peptides at 1% FDR with a 10-min retention time generated after database searching of previous LCMS runs (as described below).

Data analysis was performed utilizing Proteome Discoverer version 1.4 (Thermo Fisher Scientific) against Human Swissprot Database version March 2015 (Swiss Institute of Bioinformatics, Switzerland). Mascot 2.3.2.0 (Matrix Science) was used as a search engine with a precursor mass tolerance of 5 ppm and a fragment mass tolerance of 100 mmu. Tryptic peptides were accepted with one missed cleavage and methionine oxidation was set as the variable modifications and cysteine alkylation as the static modification. The detected peptide threshold in the software was 1% False Discovery Rate by searching against a reversed database, and identified proteins were grouped by sharing the same sequences to minimize redundancy.

### Immunofluorescence microscopy

The seeded cells after treatment were wash and fixed with 3.7% paraformaldehyde at room temperature for 10 min, permeabilized for 5 min with 0.2% Triton X-100, and washed and blocked for 1 h in 3% BSA. Cells were stained with primary antibody for 1 h and washed with PBS for three times. Finally, incubation with GFP-AF-488 antibody (1:200, A21311, Life Technology) was performed for 1 h at RT. After three washes with PBS, the cells were further stained with DAPI (Sigma Aldrich) and coverslips were mounted using Gold anti-fade mounting reagent (Invitrogen, Carlsbad, CA, USA) and observed under a fluorescence light microscope (Axio Observer, Zeiss, Oberkochen, Germany). The above-mentioned staining protocol was also performed to stain MSCs for nuclear SMAD2 (s-20, Sc-6200, Santa Cruz Biotechnology, CA, USA) after exosome treatment, Nuclear expression of SMAD2 was evaluated with the Velocity image analysis software (PerkinElmer, Chicago, IL, USA).

### Transmission electron microscopy

To describe the nano-structures in exosomes derived from primary human mast cells, we performed transmission electron microscopy (TEM). Isolated exosomes described in the section “Isolation of exosomes (i)” were bottom loaded and floated on an iodixanol (OptiPrep®) density gradient (0%, 20%, 22%, 24%, 26%, 28%, 30%, and 50%) and centrifuged at 182,300 × *g* for 16 h (SW40-Ti Rotor) to separate them from free proteins. Nine different iodixanol fractions were collected, and samples from fraction no. 2 were subjected to TEM as described in our previous study [57].

### Labelling of exosomes and uptake

Exosomes were obtained as described in the “Isolation of exosomes (i)” section and were labelled with the PKH67 Green Fluorescent Cell Linker Kit (Sigma Aldrich) as per the manufacturer’s protocol and as described previously [57] with modifications in the removal of unbound dyes. The labelled exosomes were loaded at the bottom of an iodixanol cushion (0%, 20%, 30%, and 50%) and centrifuged at 182,300 × *g* for 4 h (SW40-Ti Rotor) to separate them from the free unbound dye. The lipid-labelled exosomes were collected from the interphase between 20% and 30% and washed in PBS and centrifuged at 120,000 × *g* for 3.5 h (Type 45 Ti rotor, Beckman Coulter). Washed exosomes were incubated with the MSCs (4,000 cell/cm^2^) for 4 or 16 h at 4°C or 37°C. FACS was performed on the cells to determine the uptake rate. For visualization, exosomes were incubated with MSCs for 4 h, fixed in 3.7% paraformaldehyde, stained with DAPI, and imaged under a fluorescence light microscope (Axio Observer, Zeiss).

### Fluorescence correlation spectroscopy

Freshly isolated exosomes from HMC-1 cells were labelled either alone with optimized concentration of DiO (Life Technologies, Thermo Fisher Scientific) lipid dye (0.22 µg/ml), TGFβ-1-Alexa Fluor-647 (1:50) and CD63-Phycoerythrin (1:20), or a combination of two labels (DiO/TGFβ-1-AF647 or TGFβ-1-AF647/CD63-PE or DiO/CD63-PE). All labelled EVs were purified from the free label using an iodixanol density cushion as described in the “Labeling of exosomes and uptake” section. Washed pellets were analyzed by dual-colour Fluorescence Cross-Correlation Spectroscopy (FCCS). Two different FCS/FCCS setups were used. The first was a confocal microscope (FCS-equipped Zeiss 780) fully equipped for FCS and FCCS measurements. On this setup, we used mainly the 488 nm and 633 nm laser lines, but to some extent also the 514 nm and the 561 nm laser lines. The 488 nm laser line resulted in a focus radius ω_0_ = 0.25 μm and a volume of 0.45 femtoliters (fL), while the 633 nm laser line gave a focus with ω_0_ = 0.29 μm and a volume of 0.65 fL. Analysis of the FCS/FCCS curves was performed using the Zeiss Zen software. In addition, a home-built FCCS setup based on a 488 nm line (Argon laser, Lasos GmbH) and a 594 nm line (HeNe laser, Laser2000 GmbH) was used. In this setup, the 488 focus had ω_0_ = 0.36 μm and a volume of 1.5 fL, while the 594 focus had ω_0_ = 0.39 μm and a volume of 2.4 fL. Emission filters ET535/70 and ET700/75 (Chroma) were used. The correlator was an ALV-5000 (ALV GmbH). Analysis was performed using the ALV-5000 software and Origin 9.1 (Origin lab Corporation, USA).

### Apoptosis induction and inhibition

HMC-1 cells were cultured in cell culture medium supplemented with either 1 mM hydrogen peroxide (Sigma) or with 20 µM Z-VAD-FMK (Invivogen, CA, USA) for 72 h before conditioned supernatant was taken for exosomes isolation as described in “Isolation of exosomes–using density cushion”.

### TGFβ-1 detection on exosomes

The amount of TGFβ-1 in the supernatant of MSCs and on the HMC-1 cell line-derived exosomes was determined using a TGFβ-1 ELISA Ready-SET-Go kit (eBioscience, Affymetrix) according to the manufacturer’s instructions. To determine the forms of TGFβ-1, we either included or did not include the acidification step (HCl) to measure total and active TGFβ-1 levels, respectively. To measure the relative level of TGFβ-1 on primary human matured mast cells on captured CD63^+^ exosomes, we used a high-sensitivity chemiluminescence-based detection system (Roche Molecular Systems, IN, USA). For this experiment, we used a CD63 antibody to coat the ELISA plate, and the remaining antibodies were from the TGFβ-1 ELISA Ready-SET-Go kit.

### Quantitative real-time polymerase chain reaction

Total RNA was isolated from MSCs using the miRCURY™ RNA isolation kit for cell and plant (Exiqon, Vedbaek, Denmark). TURBO™ DNase treatment and removal reagents (Ambion, Life Technologies) were used to remove contaminating DNA from RNA preparations, and the concentration and purity of RNA were evaluated by NanoDrop (Thermo Scientific). cDNA was synthesized from 200 ng total RNA by using the iScript™ cDNA Synthesis Kit (BioRad) according to the manufacturer’s protocol. Quantitative real-time PCR was performed with SsoAdvanced™ Universal SYBR® Green Supermix on a BioRad CFX96™ system. cDNA was denatured for 30 s at 95°C and then subjected to 40 cycles of 95°C for 15 s and 60°C for 30 s. The primers were obtained from Sigma (KiCqStart® primers): TGFB1, SMAD2, MMP2, and EF1. Data were collected by software and analyzed. EF1 was used to normalize the data, and the 2_T_^−ΔΔC^ method was used to determine relative changes in gene expression.

### Western blotting

MSCs were lysed in RIPA buffer (Cell Signaling Technology ST, Danvers, MA, USA), 1 mM PMSF, 1 mM Sodium orthovanadate, and 1 mM Sodium fluoride. Twenty micrograms of protein lysate were subjected to SDS-PAGE and transferred onto nitrocellulose membranes. Membranes were blocked with 5% non-fat milk (or 5% bovine serum albumin for phospho-antibodies) in TBS containing 0.05% Tween-20 and incubated with primary antibodies at 4°C overnight and HRP-conjugated secondary antibodies (1:10,000 dilution, NA931V, NA9340, NA9310V, GE Healthcare) for 1 h at room temperature. The proteins were detected with the ECL Prime Western Detection (GE Healthcare) according to the manufacturer’s protocol. The antibodies used were as follows: β-actin (1:10,000 dilution, 13E5, #4970S, Cell Signaling Technology), TSG101 (1:1,000 dilution, ab83, Abcam), CD81 (1:1,000 dilution, sc9158, Santa Cruz Biotechnology), EEA1 (1:1,000 dilution, sc33585, Santa Cruz Biotechnology), LAMP1 (1:1,000 dilution, ab24170, Abcam), FLAG-antibody (1:5,000 dilution, #M2, F3165, Sigma), Flotillin-1-antibody (1:1,000 dilution, #sc74566, Santa Cruz Biotechnology), pSMAD2 (1:500 dilution; Thr220; #sc135644; Santa Cruz Biotechnology), SMAD2 (1:1000 dilution; S20 ; Santa Cruz Biotechnology), and 6x-His Tag (1:1,000 dilution; #MA1-21315-A488;  HIS.H8 Thermo Scientific).

### Particle number and size measurements

The sizes and concentrations of exosomes were measured using ZetaView® PMX110 (Particle Metrix, GmbH, Meerbusch, Germany). Each exosomes sample was diluted in PBS in a range of 1:1,000 ~ 1:5,000 and injected into the instrument. The chamber temperature was maintained automatically. Measurements were obtained in triplicate, and each individual result was obtained from two stationary layers with five measurements in each layer. The sensitivity of the camera was fixed at 70 in all measurements. Data were analyzed using ZetaView analysis software version 8.2.30.1 with a minimum size of 5 nm, a maximum size of 1,000 nm, and a minimum brightness of 20.

### Lineage differentiation of MSCs

MSCs treated with 100 μg/ml exosomes for 48 h were cultured in lineage differentiation medium for 15 days as per the manufacturer’s instructions (Human mesenchymal stem cell functional identification kit, SC006, R&D Systems). Lineage-specific markers for adipocytes (FABP4, Oil red O) and osteocytes (Osteocalcin, Alizarin Red) were probed and analyzed in the respective cells as described in previously published guidelines [60].

### TGFβ-1 knockout

Stable doxycycline-regulated Cas-9-expressing human mast cells (HMC-1) were generated by cloning a pCW-Cas9–containing plasmid (Gift from Eric Lander and David Sabatini; Addgene Plasmid #52961). Target gRNA (ACCAAAGCAGGGTTCACTAC) against the human *TGFbeta-1* gene was cloned into the plasmid backbone of pLX-sgRNA (Gift from Eric Lander and David Sabatini (Addgene Plasmid #50662)) with Reverse (R1) primer 5ʹ-GTA GTG AAC CCT GCT TTG GTC GGT GTT TCG TCC TTT CC-3ʹ and Forward (F2) primer 5ʹ-GAC CAA AGC AGG GTT CAC TAC GTT TTA GAG CTA GAA ATA GCA A-3ʹ.

The gRNA was chosen from library B of the Human GeCKO v2 Library (2-Plasmid System – lentiGuide-Puro; cat #1000000049). The pLX-TGFB1 gRNA-carrying plasmid was expanded and purified from chemically competent *Escherichia coli* (MAX Efficiency® Stbl2, Life Technology) and transfected into Cas-9-HMC-1 cells with doxycycline (0.5 μg/ml). Knockout clones were selected using limiting dilution of cells under blasticidin. Efficiency of knockout was tested by estimating the level of TGFβ-1 protein in knockout cell lysate (Supplementary Figure 10).

### Localization of exosomes and isolation of organelles

Isolated exosomes (as described in the method section under “Isolation of exosomes”) were loaded at the bottom of an iodixanol gradient (0%, 20%, 22%, 24%, 26%, 28%, 35%, and 50%) and centrifuged at 182,300 × *g* for 16 h (SW40-Ti Rotor) to purify the exosomes from the 20–22% layer of the iodixanol gradient. The surfaces of the iodixanol-purified exosomes (10 μg/ml) were biotinylated by incubating them with EZ-Link Sulfo-NHS-Biotin (Thermo Scientific) as per the manufacturer’s recommendation. Free biotin was removed from biotinylated exosomes via dialysis by sequentially changing PBS after 2 h (room temperature), overnight (4°C), and finally for 2 h (room temperature). These biotinylated exosomes were later incubated with HEK293T cells for 60 min, and organelles were isolated from these cells using a lysosome enrichment kit (Thermo Scientific) following the manufacturer’s guidelines with further modification to enrich lysosomes and endosomal compartments from the organelle pool. Briefly, 100 mg HEK293T cells were mixed with reagent “A” with short vortexing followed by lysis via sonication pulses (25–30 bursts, 2 min each, 4°C). This suspension was mixed uniformly with an equal volume of reagent “B” and centrifuged (500 × *g*, 10 min, 4°C) to collect the supernatant. This supernatant was loaded onto the top of an iodixanol density gradient (17%, 20%, 23%, 27%, and 30%) and centrifuged at 145,000 × *g* for 2 h at 4°C. After the first round of ultracentrifugation, the first two fractions (green and red, Figure 5(c)) were collected and bottom loaded in two separate tubes with different iodixanol densities (18%, 20%, 22%, 22%, 24%, 26%, 28%, 30%, 35%, and 50%) for the second round of ultracentrifugation (182,300 × *g*, SW40-Ti Rotor, 16 h, 4°C). The fractions were collected from top to bottom (1 ml each for 10 factions) and subjected to western blotting analysis of lysosome (LAMP1) and endosome (EEA1) enrichment markers to define the distribution of the density. From these fractions, the lysosome-enriched fraction (LEF; fraction 2–3) and the endosome-enriched fraction (EEF; fraction 7–9) were used to locate the biotinylated exosomes (Figure 5(d)).

In order to locate the biotinylated exosomes the level of TGFβ-1 protein inproteins in these fractions (LEF and EEF), two complementary approaches were used. First, LEF and EEF were both incubated separately with streptavidin-coated beads and probed for total bound protein or EEA1 and LAMP1 antibody (Supplementary Fig 12). In the second approach, LEF and EEF fractions were both incubated separately either with EEA1 or LAMP1-coated beads and probed to detect biotinylated proteins in a respective fraction (Figure 5(f)).

### SMAD3 phosphorylation assay

Activation of SMAD3 in exosomes-treated MSCs was detected by using Homogeneous Time Resolved Fluorescence (HTRF®)-based detection of phosphorylated SMAD3 at the Ser423/425 residues using the Cisbio Bioassay kit (Codolet, France). Briefly, 15,000 MSCs were seeded in a 96-well plate overnight. The cells were then serum deprived for 2 h before any treatments. Subsequently, the cells were exposed to either exosomes derived from mast cells (30 µg/ml) or free TGFβ-1 (10 ng/ml) for 30 min. The cells were then washed and lysed, and the estimation of pSMAD3 was performed according to the Cisbio Bioassay guidelines (Part no #63ADK025PEG).

### Removal of surface TGFβ-1

TGFβ-1 present on the surface of exosomes was removed by treating the crude exosomes (obtained with the iodixanol cushion) with either 0.125% trypsin (Hyclone Laboratories, Logan, UT, USA) or 30 U/ml of Bacteroides heparinase II (New England Bio Labs, Inc. USA) to remove surface protein or surface heparin and heparin sulfate, respectively. These samples were bottom loaded and refloated on an iodixanol density gradient as described in “Isolation of exosomes: using density cushion ” and analyzed for the presence of TGFβ-1 (total and active) and CD81. To inhibit cellular glycosaminoglycan, we treated HMC-1 cells with 1 mM of the pharmacological inhibitor p-nitrophenyl-beta-D-xylopyranoside (pNP-Xyl) for 72 h, and exosomes were isolated from the conditioned medium.

### In vitro interaction of LAP with active-TGFβ-1 and cellular uptake

Interaction of Latent Associated Peptide (LAP) with active-TGFβ-1 was determined by the ability of LAP-TGFβ-1 complex to migrate during gel-electrophoresis in non-reducing condition. Briefly, 200 ng of recombinant LAP protein (#246-LP, R&D system) was incubated with 50 ng, 100 ng and 200 ng of active-TGFβ-1 (TGFβ-1-Standard; component of TGFβ-1 ELISA kit; eBioscience, Affymetrix) for 30 min at 37°C. This complex was allowed to separate on PAGE and the gel was stained with silver staining reagents as per manufacturer instruction (Pierce™ Silver Stain Kit # 24612, Thermo Fisher Scientific). To determine the cellular uptake of LAP by MSC cells, we first incubated 1000 μg of HMC-1 derived-exosomes with 10 μg of Histidine-tagged recombinant human LAP (TGFβ-1) (#LAP-H5245, Acrobio systems) for 60 min at RT. This complex was loaded at the bottom of iodixanol density gradient (10%, 24%, 30%, and 50%) and centrifuged overnight at 182,300 ×g for 16 h using an SW40-Ti rotor. Interphase exosomes were collected from fractions between layers 10%-24% and was used for uptake analysis. The localization of histidine tagged human LAP (TGFβ-1) in MSCs was determined by anti-histidine antibody-AF488 (#MA1-21315-A488, Invitrogen) using fluorescence microscope.

### In vitro exosome acidification assay

HMC-1–derived exosomes from 150 ml conditioned medium were subjected to different pH conditions (pH = 7, 6, 5, 4) for 60 min at 37°C. These preparations were used to collect EVs by re-centrifuging at 52,000 rpm for 60 min in a TLA-100.3 rotor (Beckman Coulter, CA, USA). The pelleted EVs were subjected to total-TGFβ-1 ELISA (Figure 6(d)) as described earlier.

### Statistical analysis

Analyses were performed using the Student’s t-test (two-tailed). Data presented are mean±sem. and P < 0.05 was considered statistically significant.

## Results

### TGFβ-1 is present on the surface of mast cell-derived exosomes

**Figure 1.**
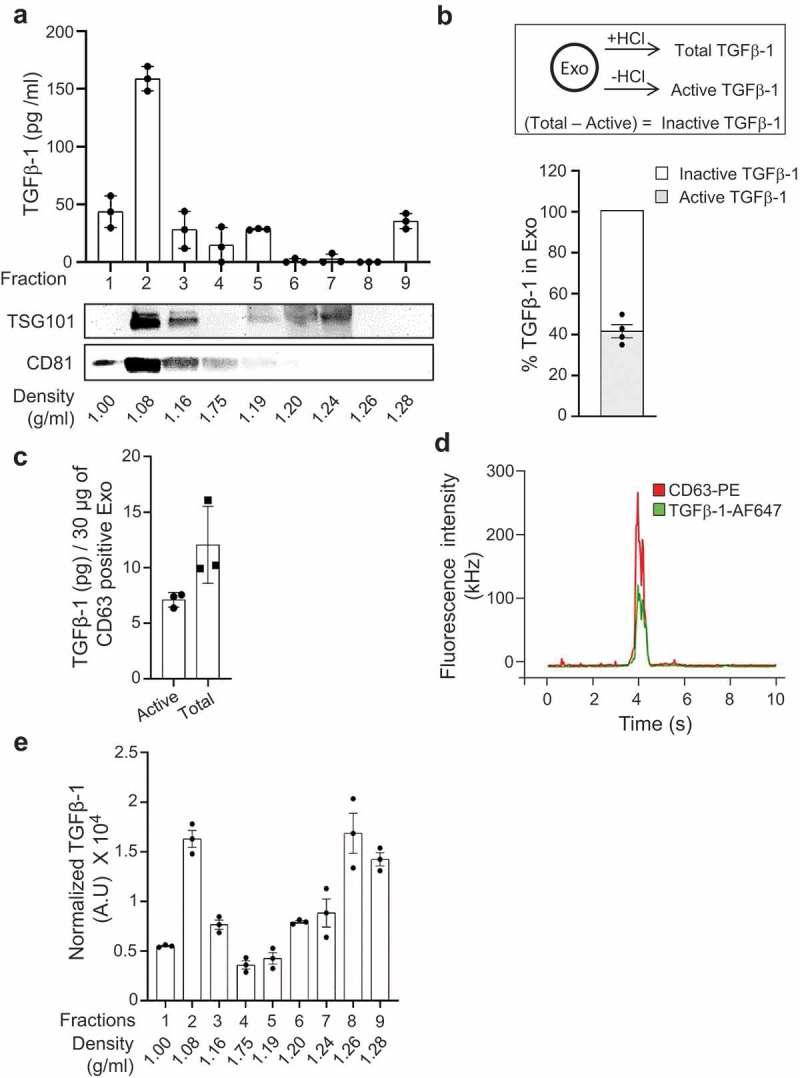
TGFβ-1 co-localizes with exosomes. (a) Exosomes isolated from HMC-1 were floated on iodixanol density gradients, and the expression of TGFβ-1 (ELISA) and the exosome-enriched proteins TSG101 and CD81 (immunoblotting) were measured (n = 3, volume = 600 ml). (b) Percentage of inactive and active forms of TGFβ-1 measured on the mast cell-derived exosomes (n = 3). (c) Amount of inactive and active forms of TGFβ-1 on the mast cell-derived exosomes was measured per unit of CD63-positive exosomes using ELISA. Data are presented as means ± SEM (n = 3). (d) Quantitative measurements of the distribution of TGFβ-1-AF647 (Red) and CD63-PE (Green) on single exosomes using fluorescence correlation spectroscopy (FCS). Excitation was at both 488 nm and 594 nm. The width of the signal, extending to more than 500 ms, corresponds to vesicle diameters. (e) Exosomes were first floated on a density gradient and then incubated on a CD63 antibody-coated on plate, and finally, the relative luminescence signal of TGFβ-1 was measured using the sandwich ELISA approach (n = 3, volume = 200 ml).

It is known that the surface and lumen of exosomes can harbour multiple bioactive molecules [25]. For example, an earlier study from our group described the presence of bioactive c-KIT receptor on mast cell-derived exosomes, and this functional receptor is transferred between cells [3]. With the intention to profile other bioactive molecules to determine the function of these exosomes, we first performed membrane proteomics of the mast cell line HMC-1-derived exosomes. This proteomics approach reduces extra- and intra-vesicular proteins, but enriches membrane proteins and can, therefore, detect various low-abundance bioactive proteins that are not detected in standard proteomics assays [26]. In total, we identified 1743 proteins (Supplementary Table 1), of which 504 were membrane-proteins, including several receptors such as TGFβ and insulin receptors. Further analysis also confirmed the presence of c-KIT receptor, supporting our previous study [3]. Interestingly, we also detected cytokines, including MIF, TIMP1, and TGFβ-1, molecules that are otherwise rarely identified with mass spectrometry of biological samples due to their low abundance (Supplementary Table 1). Multiple approaches were taken to validate the presence of TGFβ-1 on the vesicles. Firstly, we used enzyme-linked immunosorbent assay (ELISA) to confirm the presence of TGFβ-1 in the vesicle-rich floating fraction of a density gradient of HMC-1 cell line-derived exosomes [density of approximately between 1.08 and 1.16 g/ml (Figure 1(a))]. The presence of TGFβ-1 was coincident with the commonly used exosome-enriched protein TSG101 and CD81 as detected with western blot (Figure 1(a), lower panels). Similarly, the floating fractions of exosomes derived from from primary mast cells also contained TGFβ-1, again shared similar density as with cell line-derived EVs (Supplementary Figure 1). However, for the primary mast cell-derived exosomes the levels of TGFβ-1 in the high-density fraction were higher than in the low-density fractions. Exosomes released by primary cells had comparable size and structure as the cell line-derived exosomes as determined by electron microscopy, nanoparticle tracking analysis and they expressed exosomes-enriched proteins such as CD63, CD81, and CD9 (Supplementary Figure 2). However, the exosomes from primary mast cells carried relatively lower levels of the exosomes-enriched surface CD81 and CD9 (Supplementary Figure 2F) than the HMC-1 cell line-derived vesicles (Supplementary Figure 2E).

TGFβ-1 exists in an active and in an inactive form, and they can be discriminated by the use of hydrochloric acid (HCl) acidification of the samples, after which TGFβ-1 ELISA is performed (Figure 1(b), upper panel). We observed that approximately 40% of the total TGFβ-1 on the exosomes was in the active form (Figure 1(b), lower panel), and 60% in the inactive form. Finally, we performed a sandwich ELISA to quantify the amount of TGFβ-1 associated with exosomes bound to anti-CD63-coated beads. We could detect approximately 12 pg of total TGFβ-1 on 30 µg of CD63-positive exosomes from HMC-1 cells, out of which 7.1 pg was in the active form (Figure 1(c)). In addition, the co-association of TGFβ-1 with an exosome was observed by fluorescence correlation spectroscopy (FCS). Specifically, we found that the signals for TGFβ-1 and the exosome-enriched surface protein CD63 were co-localized in the same time lapse and had the same diffusion time in the FCS analysis (Figure 1(d)), which strongly suggests that they are co-localized on the same individual vesicle. With this method, we determined that approximately 17% of the CD63+ vesicles were also positive for surface TGFβ-1. Additionally, we used sandwich ELISA to detect the levels of TGFβ-1 on CD63+ vesicles using capture antibody against CD63 in various HMC-1 fractions after iodixanol density gradient separation (Figure 1(e)).

To exclude the possibility that TGFβ-1 was associated with the exosomes mechanically through the isolation procedure (ultracentrifugation), we also used a non-pelleting approach for exosomes isolation. Specifically, exosomes were collected in the interphase between 10% and 30% after an iodixanol density cushion centrifugation and were further floated on a density gradient and collected at ~1.08 g/ml density (Supplementary Figure 3a). Even though the viability of the HMC-1 cells used for exosomes isolation was >99% using a trypan blue assay, we further investigated the possibility of background apoptosis contributing to the presence of TGFβ-1. Briefly, the HMC-1 cells were treated with an apoptosis inhibitor (Z-VAD-FMK) or inducer (H_2_O_2_) and exosomes were collected using the non-pelleting approach (Supplementary Figure 3a, b). When apoptosis was induced (H_2_O_2_), an increased number of particles could be isolated; however, no significant increase in exosome-associated TGFβ-1 was observed (Supplementary Figure 3c-d). Similarly, when apoptosis was inhibited (Z-VAD-FMK), no significant reduction of exosomes-associated TGFβ-1 was observed (Supplementary Figure 3d). Furthermore, the presence of surface CD9, CD63, and CD81 along with luminal flotillin-1 was confirmed in TGFβ-1–captured vesicles  (Supplementary Figure 3e-f). Taken together these experiments conclusively show that TGFβ-1 is associated with exosomes on their surface and is not introduced to the exosomes during the ultracentrifugation or by cell death.

### Glycan-based association of inactive TGFβ-1 with exosomes

**Figure 2.**
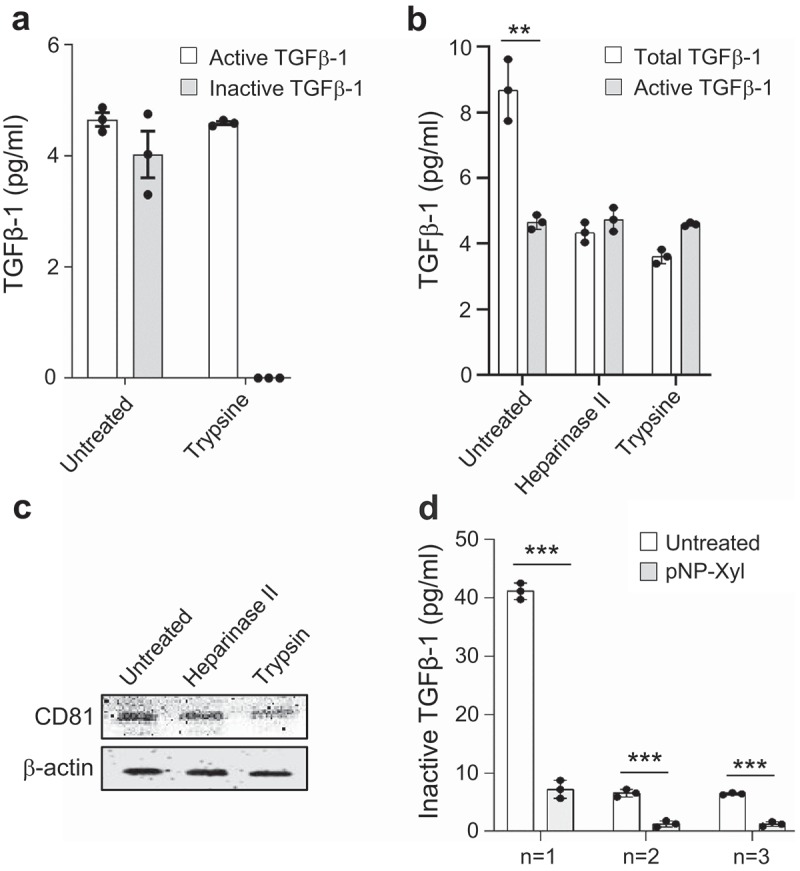
Glycan-based association of inactive TGFβ-1 on exosomes. The presence of TGFβ-1 was evaluated on exosomes isolated with a pelleting-free protocol from HMC-1 cells (see Supplementary Figure 3A). (a) Presence of TGFβ-1 on exosomes treated with trypsin (0.125%) measured by ELISA. (b,c) Exosomes treated with Heparinase-II and trypsin and re-floated were evaluated for the presence of TGFβ-1 and the presence of surface (CD81) and luminal (β-actin) markers on exosomes (n = 3, volume = 100 ml). (d) Level of TGFβ-1 on exosomes after competitive inhibition of glycan biosynthesis by the addition of pNP-Xyl (n = 1, volume = 600 ml; n = 2, 3 volume = 200 ml).

To determine how the different forms of TGFβ-1 are associated with the exosomes surface, we first obtained purified exosomes with the non-pelleting approach (Supplementary Figure 3a). Exosomes were treated with trypsin (0.125%) and again floated on a density gradient. The inactive form of TGFβ-1 was efficiently removed by the trypsin treatment, while the active form remained associated with the exosomes (Figure 2(a)). Interestingly, the active form of TGFβ-1 could very well be associated with the TGFBR1 that we identified earlier in membrane proteomics analysis (Supplementary Table 1). Indeed, we confirmed our findings from the exosome membrane proteomics by immunoblotting analysis of exosomes of TGFBR1, a known ligand of active-TGFβ-1 (Supplementary Figure 4).

Inactive TGFβ-1 contains the latent-associated peptide (LAP), and it is known that the LAP-TGFβ-1 complex can associate with the extracellular matrix by binding to heparan sulfate glycoprotein (HSPGs) [27,28]. To determine whether HSPGs such as heparin and heparan sulfate are important for the association of LAP-TGFβ-1 to the exosome-surface, exosomes were treated with heparinase-II. When exosomes were treated with heparinase-II, only inactive-TGFβ-1 was removed from the exosome, and no significant alterations were observed in the active-TGFβ-1 levels (Figure 2(b)). Additionally, trypsin treatment slightly reduced the surface protein on the exosome surface (CD81) compared to the luminal β-actin indicating that the EVs remain intact during the treatments (Figure 2(c)). However, heparinase-II does not have an effect on any membrane and luminal exosomes (Figure 2(c)). HSPGs are present on the outside of the cell membrane, and HSPGs such as syndecan and glypican have been reported to be markers of EVs [29,30]. To determine whether the association of HSPGs with TGFβ-1 is related to active cellular proteoglycan synthesis, we treated the HMC-1 cells with the inhibitor p-Nitrophenyl-β-D-xylopyranoside (pNP-Xyl). Exosomes isolated from pNP-Xyl-treated cells showed a significant decrease in total TGFβ-1, indicating a novel mechanism by which TGFβ-1 can be associated with the surface of exosomes (Supplementary Figure 5a). We then evaluated whether the reduction seen is due to the change in inactive or active TGFβ-1. Exosomes isolated from pNP-Xyl treated showed a lowering of inactive TGFβ-1 with no significant changes in active TGFβ-1 (Figure 2(d), Supplementary Figure 5b). Taken together these findings suggest that the inactive form of TGFβ-1 is associated with exosomes via heparin and heparan sulfate sensitive glycoproteins.

### Mast cell-derived exosomes enhance the migratory function of MSCs

**Figure 3.**
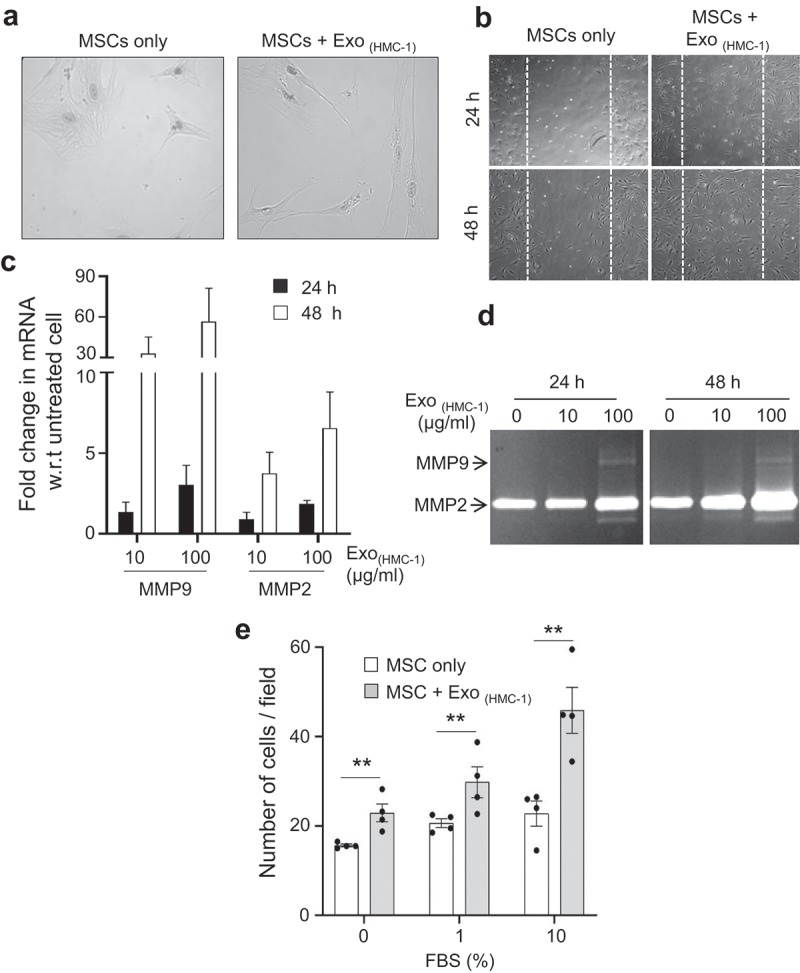
Mast cell-derived exosomes enhance the migration of primary human mesenchymal stem cells. (a) Morphology of MSCs incubated with exosomes derived from HMC-1 cells. (b) An *in vitro* wound healing scratch assay was performed on monolayers of untreated or exosomes-treated MSCs (n = 2) that were derived from HMC-1 cells. MSCs were imaged at 24 h and 48 h after injury. (c) Expression of MMP-9 and MMP-2 transcripts in MSCs after exosome treatment using quantitative polymerase chain reaction. w.r.t; with respect to. (d) Gelatinolytic activity in secreted supernatant from MSCs estimated by zymography to detect MMP-2 and MMP-9 activity. (e) The migration activity of the exosome-treated MSCs towards different concentrations of FBS evaluated using a Boyden chamber migration assay. Data are presented as means ± SEM; n = 4; ** p ≤ 0.01.

During inflammation, immune cells are known to modulate the phenotype of recipient cells by various secreted factors, including exosomes [31,32]. We hypothesize that exosomes from mast cells induce phenotypic changes in human MSCs. HMC-1 exosomes were efficiently taken up by primary human MSCs in a time- and temperature-dependent manner, which suggests that vesicle uptake is a biologically active process (Supplementary Figure 6). Adding exosomes derived from HMC-1 as well as primary mast cells to the MSCs induced an elongated morphology in the MSCs (Figure 3(a), Supplementary Figure 7a) and increased wound healing activity (Figure 3(b), Supplementary Figure 7b) but did not alter the ability of the MSCs to differentiate because they still could differentiate into both adipocytes and osteocytes (Supplementary Fig. 8). Enhanced migratory activity of MSCs upon exosomes stimulation was also associated with the increase in transcripts of matrix metalloproteinase (MMP-2 and MMP-9) mRNA in cells and with increased secretion of MMP-2 and MMP-9 proteins into the cell culture supernatant and was associated with the increased gelatinolytic activity (Figure 3(c,d)). It has previously been shown that multiple growth factors can influence MSC migration by regulating signalling cascades [33]. We therefore used fetal bovine serum (FBS) as a pan-chemoattractant in the next experiment and could show that exosome-treated MSCs have an increased and dose-dependent migratory tendency towards the FBS compared to non-treated MSCs (Figure 3(e)). Collectively, these results show unequivocally that mast cell-derived exosomes enhance the migration of MSCs *in vitro*.

### TGFβ-1 on the surface of exosome induces activation of SMAD in MSCs

**Figure 4.**
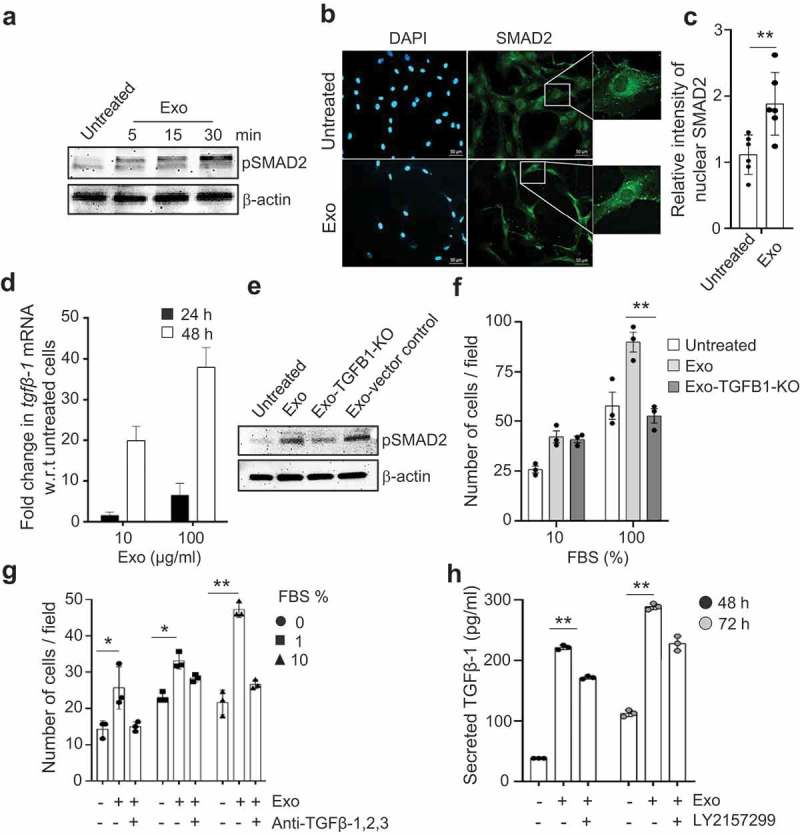
Exosomes activate SMAD signalling in human mesenchymal stem cells. (a) Exosomes (100 µg/ml) activate the TGFβ-1 signalling axis by phosphorylation of SMAD2 as identified by immunoblotting at different time points. (b,c) Activation is visualized by immuno-fluorescent imaging of the translocation of SMAD2 into the nucleus (b) and is quantified by the relative intensity measurement (c) in MSCs 30 min after exosome exposure. (d) Activation of transcripts downstream of SMAD2, such as TGFB1, was evaluated in MSCs at 24 h and 48 h after exosome exposure. (e,f) Exosomes from TGFβ-1 knockout HMC-1 cells were used to induce phosphorylation of SMAD2 in MSCs as measured using immunoblotting (e) and as measured by their capacity to induce migration towards FBS (f). (g) Migration capacity of MSCs towards FBS after exosome treatment was also evaluated in the presence of a blocking antibody against TGF-β-1, 2, 3 using a Boyden chamber migration assay. (h) Secretion of TGFβ-1 in MSCs was measured in the presence of the TGFR-1 receptor blocking agent LY2157299 during exposure to exosomes at 24 h and 48 h. Data are presented as means ± SEM; n = 3; * p ≤ 0.05, ** p ≤ 0.01.

It has earlier been described that soluble TGFβ-1 can stimulate MSC migration [34], and because we observed that TGFβ-1 is present on the membrane of exosomes, we set out to determine whether this molecule is responsible for the observed migration activity of the cells. Importantly, SMADs are a family of proteins that are involved in signal transduction and transcriptional regulation [35], and SMAD2 is activated in response to TGF-β signalling and might be involved in MSC migration [36]. Indeed, we observed increased phosphorylation of SMAD2 in MSCs after exosome exposure (Figure 4A). Furthermore, the ratio between nuclear and cytoplasmic SMAD2 in MSCs increased following exosomes treatment, indicating that exosomes induced nuclear translocation of this transcription factor (Figure 4(b,c)). To determine whether the signalling is restricted to SMAD2, or whether any other SMADs were regulated, we determined the phosphorylation of SMAD3 using a fluorescence resonance energy transfer (FRET) based assay in recipient MSCs (Supplementary Fig. 9). Consistent with activation of SMAD2, we also observed a time- and dose-dependent increase in *Tgfb1* mRNA transcript following exposure to exosomes (Figure 4(d)). This suggests that exosomes enhance *Tgfb1* expression in MSCs, most likely via SMAD2 signalling. This is consistent with a previous study documenting that soluble TGFβ-1 regulates its own expression in an autocrine manner [37].

To determine whether exosome-associated TGFβ-1 is responsible for the observed enhanced migration and TGFβ-1signaling in MSCs, we first isolated exosomes from TGFB1 knockout HMC-1 cells (doxycycline-inducible CRISPR/Cas-9 system; Supplementary Fig. 10a-b). Indeed, exosomes derived from TGFβ-1 knockout mast-cells resulted in reduced phosphorylation of SMAD2 in recipient MSCs compared to exosomes from control cells (Figure 4(e)), as well as reduced migration towards the pan-chemoattractant FBS (Figure 4(f)). To further confirm the role of exosomes-surface TGFβ-1, we pre-incubated exosomes with a pan-TGFβ-neutralizing antibody that blocks the TGFβ-1, 2, 3 interaction with its receptor. The TGFβ-neutralized exosomes induced a significant reduction in the migration of the MSCs towards the pan-chemoattractant FBS (Figure 4(g)). In addition, we found a time-dependent significant reduction in exosome-induced TGFβ-1 secretion into the growth media by the MSCs when the TGFβ-1 receptor was blocked by a specific small-molecule inhibitor (LY215799) (Figure 4(h)). Collectively, these results strongly support the idea that TGFβ-1 on the exosome-surface induces an enhanced migration phenotype and TGFβ-1 response in MSCs.

### Sustained TGFβ-1 signalling by endosomal retention of exosomes and lysosome evasion

**Figure 5.**
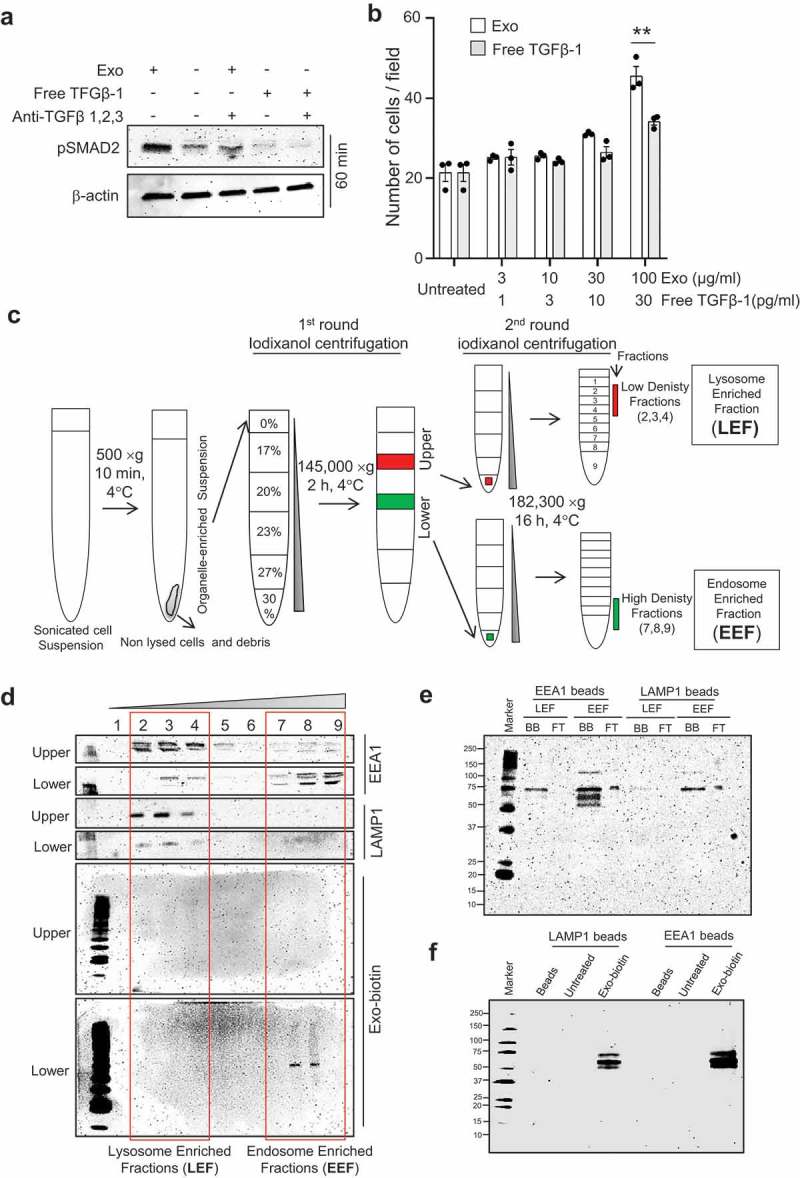
TGFβ-1 associated with exosomes has higher signalling stability compared with free TGFβ-1. (a) The signalling efficiency in MSCs, as determined by immunoblotting of pSMAD2, in response to dose-matched concentrations (10 pg/ml) of free TGFβ-1 or EV-associated TGFβ-1 estimated at 60 min. (b) The migration of MSCs towards free TGFβ-1 or EV-associated TGFβ-1 as a chemoattractant was performed by reverse migration assay. Data are presented as means ± SEM; n = 3; * p ≤ 0.05. (c) Outline of the procedure to enrich endosomes and lysosomes from cells using differential density centrifugation. Briefly, a total of 100 mg of cells was treated with biotinylated exosomes (100 μg/ml) for 60 min, after which the cells were lysed and centrifuged to remove cells and debris. Supernatants were top loaded on an iodixanol density gradient followed by a second round of iodixanol density gradient separation of the upper and lower fractions obtained from the first round of iodixanol density gradient. The final fractions for the endosome-enriched fraction (EEF) and the lysosome-enriched fraction (LEF) were collected for subsequent experiments. (d) Biotinylated EVs were incubated with HEK293T cells for 60 min, and organelles were isolated with an iodixanol gradient as described in outline and probed to detect LAMP1, EEA1, and biotinylated-exosome distribution. (e) Organelles isolated with iodixanol gradient from HEK293T treated with biotinylated exosomes followed by LAMP1 or EEA1 antibody-based capture and probed for biotinylated exosome-proteins. (F) The presence of biotinylated exosomes proteins at 60 min was confirmed in the organelle-enriched suspension of MSCs cells that were bound to LAMP1 or EEA1 antibody-coated beads.

It is well known that TGFβ-1 rapidly activates cell signalling by phosphorylation of SMADs, which is rapidly reduced within 30 min [38,39]. Having observed similar signalling activation and functional outcome for exosome-associated TGFβ-1, we asked whether free TGFβ-1 induces similar temporal signalling activation in MSCs in our system. To test this, we used dose-matched concentrations of TGFβ-1 in its free form and in the exosome-associated form and performed immunoblotting analysis for phosphorylated SMAD2 (pSMAD2) in the recipient MSCs. First, we determined the amount of TGFβ-1 associated with our total exosomes isolates after floating them on a density gradient. We found approximately 18.5 pg of total TGFβ-1 in 30 µg isolated exosomes, out of which approximately 40% was in the active form of TGFβ-1. Hence, 0.3 pg of active TGFβ-1 was present per microgram of total exosomes, and therefore this was utilized for further experiments. Interestingly, TGFβ-1 induced a different degree of activation at different time points when associated with exosomes, producing a lower degree of phosphorylation of SMAD2 at 30 min (Supplementary Fig. 11) but with more sustained effects at 60 min (Figure 5(a)). The difference in kinetics invited us to investigate of the delayed TGFb-1 signalling via exosomes provide any advantage over transit activation of free-TGFβ-1. We further observed that exosomes stimulated MSC migration more efficiently than free TGFβ-1 at dose-matched concentrations (≥30 pg/ml) (Figure 5(b)). These data collectively suggest that the sustained low amplitude signalling elicited by exosome-associated TGFβ-1 evokes greater functional effects than the transient, high amplitude signal evoked by free TGFβ-1.

To elucidate the difference in the signalling efficiency of free versus exosome-associated active TGFβ-1, we assessed the fate of exosome in intracellular compartments, primarily comparing lysosomes and early endosomes. It has previously been reported that free TGFβ-1 is rapidly processed via lysosomes [40], and we hypothesized that exosome-associated TGFβ-1 that is taken up by recipient cells evades or is delayed in trafficking to lysosomes, producing a prolonged intracellular TGFβ-signal. Because it is not feasible to perform these experiments at large scales using MSCs, we instead utilized HEK293T cells. Briefly, a high-resolution fractionation of cell organelles was performed to physically separate lysosomes and early endosomes derived from HEK293T cells that had been exposed to biotinylated-exosomes for 60 min (Figure 5(c) and (d), bottom two blots). Furthermore, we detected endosome (EEA1) and lysosome (LAMP1) markers in the different density gradients (Figure 5(d), top four blots). The respective markers were used to identify the endosome-enriched fraction (EEF) and the lysosome-enriched fraction (LEF). We observed that the EEF was highly enriched in biotinylated proteins as compared with the LEF, suggesting that the components of exosomes were at least partly taken up by the cells and were retained in endosomes at this time point (Figure 5(d), bottom two blots). The observed prolonged exosome-associated TGFβ-1–mediated activation of cells might therefore at least partly be explained by lysosomal evasion of the exosome.

To confirm the endosomal localization of the vesicle components, two different approaches were taken. First, EEF and LEF samples were incubated with organelle-specific antibodies to pull down the endosomal and lysosomal fractions (Supplementary Fig. 12, right panel) again showing enrichment of biotinylated proteins in the EEA1-specific pull-down (as a marker of endosomes) compared to the LAMP1-specific pull-down (as a marker of lysosomes) (Figure 5(e)). To confirm this finding in human MSCs, we traced the biotinylated exosomes by performing a similar organelle-specific pull-down of EEA1 and LAMP1 from crude cytoplasmic preparation of MSCs. As for the HEK293T cells, MSCs also showed a higher biotinylation signal in the endosomal vs. the lysosomal compartments (Figure 5(f)). Secondly, we also looked for endosomal traces in streptavidin-captured exosome biotin to validate the finding. HEK293T cells were incubated with biotinylated exosomes, and streptavidin-coated beads were used to pull down the cellular compartments containing biotinylated proteins, where the biotinylated exosomes proteins are present, as described in supplementary Fig. 12 (Left panel). As expected, the total amounts of proteins binding to the beads were significantly higher in the EEF compared to the LEF sample (Supplementary Fig. 12 (i)). Furthermore, a higher percentage of the proteins was bound to the streptavidin-coated beads in the EEF samples, while a higher percentage of the proteins was unbound to the beads in the LEF sample, indicating that more biotinylated proteins, and thus exosomes, were present in the endosome-enriched fraction. To further confirm that the bead-bound material (biotinylated proteins) originated from the organelles of interest, we identified the early endosomal protein (EEA1) and lysosomal (LAMP1) (Supplementary Fig. 12 (ii)) in the bead-bound samples. In summary, the results of these collective experiments show that the exosomes remained in the early endosomes, suggesting early avoidance of traffic of the exosomes to the lysosomes and thus allowing for prolonged TGFβ-1 signalling in the recipient cells.

### Exosomes assist in the uptake of inactive TGFβ-1 into the endosomal compartment

**Figure 6.**
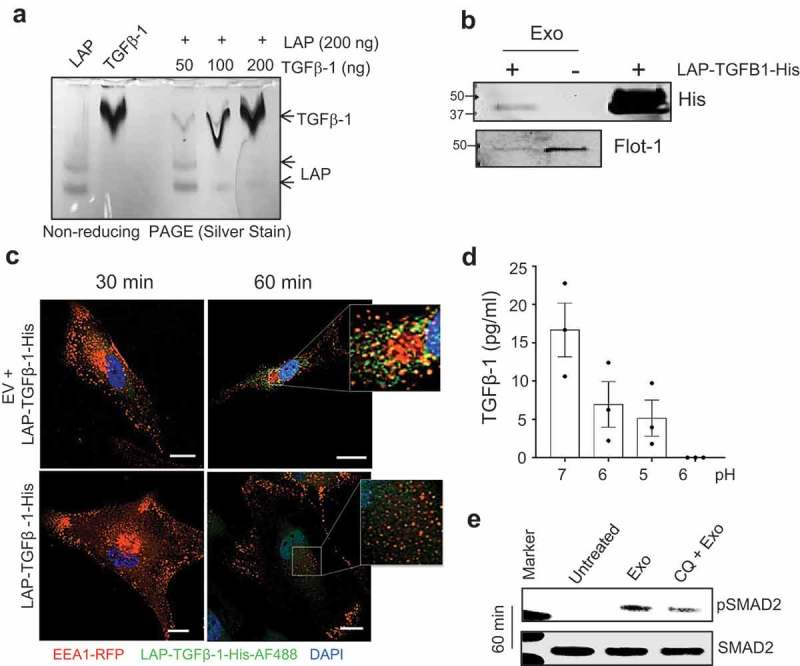
Inactive TGFβ-1 associates with mast cell-derived exosomes and uptake by an endocytic compartment of human mesenchymal stem cells. (a) *In-vitro* interaction of latent-associated peptide (LAP) and the active-TGFβ-1 complex was visualized using silver staining of native-PAGE gels. (b) Determination of *in-vitro* interactions between EVs and histidine-tagged LAP-TGFβ-1 complex with immunoblot detection of histidine and flotillin-1 on the complex that was floated on an iodixanol density gradient (10–24%). (c) Immunofluorescence detection of LAP-TGFβ-1-His with anti-histidine-AF488 antibody and endosomes (EEA1-RFP) in MSCs with or without exosomes. (d) Measure of TGFβ-1 in exosomes pellet that was treated with various pH conditions. (e) Detection of pSMAD2 using immunoblotting in MSCs that were exposed to EVs (50 μg/ml). MSCs were pre-treated with an inhibitor of endo-lysosomal acidification (chloroquine, CQ).

Presence of exosomes in the endosomal compartment does not guarantee the presence of TGFβ-1. We also know that LAP actively binds to the active form of TGFβ-1 as demonstrated *in-vitro* (Figure 6(a)). To determine the interaction of LAP-associated TGFβ-1 with exosomes *in-vitro* we incubated exosomes with recombinant human LAP-TGFβ-1 tagged with histidine (LAP-TGFβ-1-His). This tagged version (LAP-TGFβ-1-His) was used to distinguish from endogenous LAP-TGFβ-1. This incubated mixture (Exosomes + LAP-TGF#x3B2;-1-His) was loaded in the bottom of the iodixanol density gradient. The samples were centrifuged, and the floated exosomes were collected from interphase of 10–24% iodixanol. Stable interaction of LAP-TGFβ-1-His and exosomes was confirmed with immunoblotting of the LAP-TGFβ-1-His along with exosome-associated protein (Flotillin-1) (Figure 6(b)).

**Figure 7.**
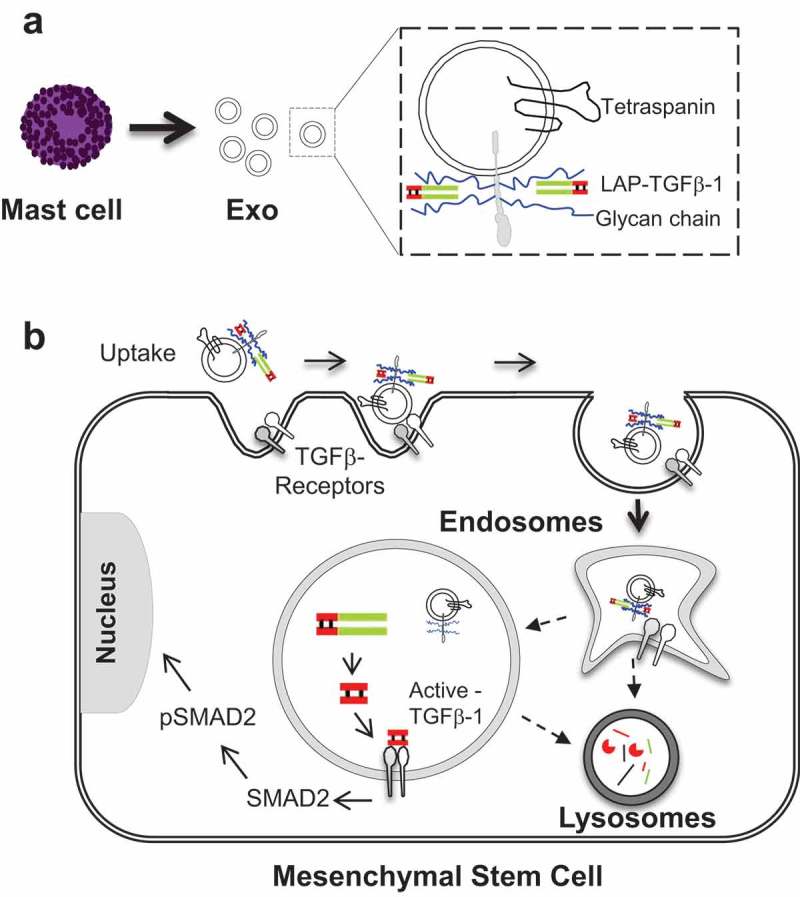
Graphical outline of the proposed mechanism. (a) TGFβ-1 and exosomes interaction and, (b) proposed mechanism of signalling in the endocytic compartment of MSCs induced by exosome-associated TGFβ-1.

This complex of exosomes and LAP-TGFβ-1-His was traced in recipient MSCs by monitoring endosomal compartments expressing red florescent protein and anti-His-Alexa Fluor-488 co-localization events. Exposure of MSCs for 60 min with exosomes and LAP-TGFβ-1-His complex showed co-localization of endosome and LAP-TGF#x3B2;-1-His (upper panel, Figure 6(c)). This co-localization was undetectable at 30 min and with LAP-TGFβ-1-His alone (lower panel, Figure 6(c)). The transition of the exosomes into the endosomal compartment may be exposed to gradual acidification, so we tested the impact of *in vitro* acidification of exosomes in *in-vitro* reaction to determine pH-dependent association of endogenous TGFβ-1 with exosomes. To our surprise, the release of TGFβ-1 from exosomes was evident from pH 6 suggesting the conversion of inactive LAP-TGFβ-1 to active TGFβ-1 is very sensitive to small changes in pH (Figure 6(d)). We then speculated as to whether acidification of the cellular compartment is essential for exosome-induced endosomal TGFβ signalling. We determine SMAD2 activation with exosome in the presence of an endosome-lysosome system acidification inhibitor (Chloroquine) and found a partial reduction in the activation of SMAD2 (Figure 6(e)). Taken together, our data indicate that acidification of the endosome-lysosome system could play an important role in exosomal--TGFβ-1-induced endosomal-signalling.

## Discussion

This study describes in detail how TGFβ-1 is associated to exosome membranes via heparinase sensitive surface glycans, dissociates from the exosome under acidic conditions, transforms from the latent to the active form, and mediates signalling in endosomes. Further, the exosomes-associated TGFβ-1 is more potent than the free form of the molecule, and we show how exosome-associated TGFβ-1 induces enhanced MSC cell-migration *in vitro* via SMAD2 signalling. We further observed that exosome traffic primarily to the endosomal compartment, and less to the lysosomes of the MSCs, at a time when SMAD2 phosphorylation is evident. Endosomal localization was evident for inactive TGFβ-1, where pH-dependent release of active TGFβ-1 from LAP could occur, which could explain the higher potency and extended biological response to exosome-associated TGFβ-1 compared to that of free TGFβ-1. Taken together, these data indicate that exosome released by human mast cells can promote MSC migration and phenotypic changes via endosomal TGFβ-1 signalling.

We and others have previously suggested that bioactive molecules on the EV surface can efficiently influence recipient cells, specifically by transfer of the stem cell factor receptor c-Kit, which might enhance recipient lung cancer cell proliferation [3,41–43]. In the current study, we have utilized an exosome-membrane proteomics approach to identify the low-abundance TGFβ-1 and its receptor (Supplementary Table 1), and we performed experiments to identify any biological functions this molecule might have. Validation of the membrane proteomics data at single-vesicle resolution confirmed the presence of TGFβ-1 on the exosomes surface (Figure 1(d)), which suggests that this highly bioactive cytokine might mediate immediate biological responses when interacting with a cell via TGFβ-1 receptor activation. Previous studies have shown that free signalling factors like platelet-derived growth factor, TGF-β, and fibroblast growth factor can induce growth and differentiation of MSCs and change the fate of MSC function, but our current study suggests that the TGF-β-1 cytokine can be more potent if associated with the exosome membrane. It is possible that this enhanced potency depends on the three-dimensional association of TGF-β-1 to the membrane, facilitating its interaction with its receptor on the recipient cell. Importantly, blocking exosomes-associated TGFβ-1 with an antibody was sufficient to significantly reduce TGFβ-signaling, further supporting the importance of this bioactive molecule for the observed biological effects in MSCs.

To validate that TGFβ-1 is specifically interacting with exosomes and to rule out that the presence of TGFβ-1 on exosomes might be mediated by centrifugation-induced aggregation, we extended the studies by using a pellet-free exosomes isolation method (Supplementary Figure 3a). Indeed, non-pelleted exosomes captured on anti-TGFβ-1 beads were expressing the tetraspanins CD81, CD63 and CD9, as well as flotillin-1, strongly arguing that the presence of TGFβ-1 on exosomes was not induced by a forceful centrifugation step (Supplementary Figure 3E-F). A detailed evaluation of the exosome-associated TGF-β-1 suggested that approximately 40% was in the active form and 60% was in the inactive form (Figure 1(b)). This distribution of TGFβ-1 was different from what was observed in a previous study by Webber et al. (2010), where they suggested that only ~2% of the TGFβ-1 from mesothelioma or prostate cancer cell line-derived exosomes was in the active form [13]. This further emphasizes that mast cell-derived exosomes might have a greater proportion of active TGFβ-1 compared to exosomes derived from other cells, which potentially could favour mast cell-derived exosomes in influencing MSC function.

This study describes the mechanism by which mast cell-derived exosomes can induce changes in MSCs to become more migratory, as shown by a Matrigel-based migration assay. The MSCs became dose-dependently more migratory after pre-conditioning with exosomes (Figure 4(f,g)). Previous approaches to treating different diseases with MSCs include pre-treatments of MSCs with cytokines such as TNF-α, IFN-γ, and IL-1β, and these have resulted in some experimental success. However, these approaches have suffered from problems like induction of MSC differentiation, loss of immune-suppressive function, and limited target usage in certain treatments [44]. Unlike other methods, MSCs pre-conditioned with mast cell-derived exosomes maintain their multipotency (Supplementary Fig. 8) and immuno-suppressive effects, which makes them interesting therapeutic candidates in diverse diseases.

In all biological systems, free biomolecules such as cytokines interact with surface receptors on cells, resulting in downstream cellular signalling [45,46]. Interestingly, picogram concentrations of TGFβ-1 on the surface of vesicles can contribute significantly to downstream signalling, for example, phosphorylation of SMAD2 (Figure 4(a)). It is thus likely that exosomes present their surface molecules to recipient cells in a unique context, which might explain the higher potency of exosome-associated TGFβ-1 compared to free molecules. Such mechanisms have indeed been suggested for exosomes in other situations; for example, they can enhance the oligomerization of prion proteins by generating high local concentrations of template and PrP^c^ proteins and bringing them into close proximity [45]. Additionally, we observed that the inactive form of TGFβ-1 is associated with exosomes via surface glycans. It is possible that the glycan-associated exosomes releases this inactive form of TGFβ-1 as active TGFβ-1 by cleavage either by cell surface proteases or upon acidification within the endosomal compartments. We observed that the association of inactive TGFβ-1 with exosomes was pH-dependent, explaining the possible conversion of inactive TGFβ-1 into its active form within endosomes (Figure 6(d,e)). Thus, emerging evidence suggests that significantly lower concentrations of bioactive molecules can induce a stronger response when associated with the exosomes, which ensures that the ligand is in close proximity to its receptor.

When comparing the effects of exosome-associated TGFβ-1 with free TGFβ-1, we could see both enhanced and prolonged biological effects of the exosome-associated cytokine, both in relation to migratory function as well as pSMAD signalling in recipient MSCs at the 60-min time point. It has previously been shown that TGFβ-1 alone induces signalling by the activation of the TGFβ-1 receptor, and this complex is rapidly internalized and is transferred to lysosomes for degradation [47,48]. Another study has shown the transfer of TGFβ-1 receptor via EV assist in SMAD2 signalling in receptor-deficient squamous cell carcinoma tumour keratinocytes [49]. Unlike receptor transfer, our current study purpose the transfer of bioactive molecules to the inside of the recipient cells. The fate of exosomes in recipient cells, which is known to involve endosomal translocation [50,51], could at least partially explain the longer signalling half-life of exosome-associated TGFβ-1 over free TGFβ-1 observed in the current experiments. To investigate this, we isolated early endosomal and lysosomal compartments at a time point where we know phosphorylation of SMAD2 was at its highest, and we observed that biotinylated exosome-proteins were preferentially located within the early endosomal compartments (EEA1-enriched), with a small amount of biotinylated exosome-proteins in the lysosomal compartment (Figure 5(c–f)) [52]. Therefore, the presence of TGFβ-1 on the surface of exosomes seems to allow its prolonged signalling, probably by avoiding degradation in the lysosomes. Additionally, *in vitro* acidification of the exosome showed that TGFβ-1 was dissociated from the exosomes already at ~pH 6, and the exosomes then induced less phosphorylation of SMAD2, further supporting that TGFβ-1 on the exosome-surface is crucial for the phenotype change of the recipient cells (Figure 6(d,e)). In this study, we suggest that inactive TGFβ-1 is attached to exosomes via HSPGs, which bring TGFβ-1 to the endosomal compartment. HSPG synthesis requires glucosamine-N-deacetylase/N-sulfotransferase-1 to add glycan chains to the glycoproteins. When this enzyme activity was diverted by adding pNP-Xyl a comparative substrate glycan, we observed a significant reduction of inactive-TGFβ-1 on the exosomes, supporting the role of glycans in binding the TGFβ-1 to the exosome-surface (Figure 2(d) and Supplementary Figure 5). This was in accordance with a previous study where endothelial cells from mouse lung showed reduced secretion of TGFβ-1, IL-5, IL-2, and Eotaxin in N-deacetylase/N-sulfotransferase-1–deficient cells [53]. Furthermore, we depleted the glycan chains (heparin and heparan sulfate) on exosomes *in vitro* with the heparinase-II enzyme, which also significantly reduced the presence of inactive TGFβ-1 on the exosomes (Figure 2(b,c)). Removal of glycan chains from the exosomes did not alter the uptake by MSCs in our experiments, which is in line with an earlier study of exosome uptake into glioblastoma cells [54]. Interestingly, an observation made by Webber, J. P *et.al* did not show any dependency of latent TGFβ-1 binding on to the exosomes via Haparinase-II sensitive elements. This could be due to the presence of high abundant anionic heparin or chondroitin sulfate proteoglycans from mast cells [55]. Interestingly, in humans, a large proportion of secretory granules is produced by mast cells and it is one of the richest sources of anionic heparin or chondroitin sulfate proteoglycans [56]. Moreover, we have a large number of HSPG (e.g. syndecan, glypican) in our exosomes preparation, as commonly reported exosomes proteomics [29,30]. Binding of TGFβ-1 to the surface of exosome via HSPGs could indeed be a novel strategy for long-term signalling by low-abundance bioactive molecules, and putative expression of other bioactive molecules on the surface of exosomes could be an important pathway by which exosomes alter recipient cell phenotypes.

Collectively, our findings suggest that exosomes from mast cells can change the phenotype and function of MSCs via TGFβ-1 bound to the surface of the exosomes via proteoglycans. In addition, the exosomes taken up by the MSCs remain in the acidifying endosomal compartments at the time of cell signalling, possibly via the pH-mediated release of latent TGFβ-1 to the active form. Further, the observed extended phosphorylation of SMAD2 by TGFβ-1 on the exosomes suggests that significant changes in potency and kinetics of responses can be observed when a bioactive molecule is present on exosomes in its free form. In conclusion, the surface of exosomes offers a scaffold for bioactive proteins binding that required endosomal environment for its activation/signalling.
