# Supplementary material for: Endosomal signalling via exosome surface TGFβ-1
Source: J Extracell Vesicles. 2019 Sep 20;8(1):1650458. doi: 10.1080/20013078.2019.1650458 (PMC6764367; doi:10.1080/20013078.2019.1650458)
Supplement: Supplemental Material [file ZJEV_A_1650458_SM9071.zip › ZJEV_A_1650458_Supplementary/MS_MSC_HMC1_EV_Proof_Supplement_Fig.pdf]

Supplementary Figure 1

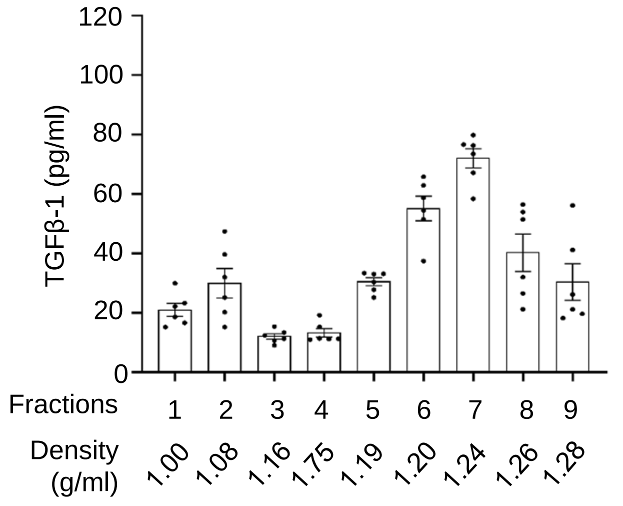

Supplementary Figure 2

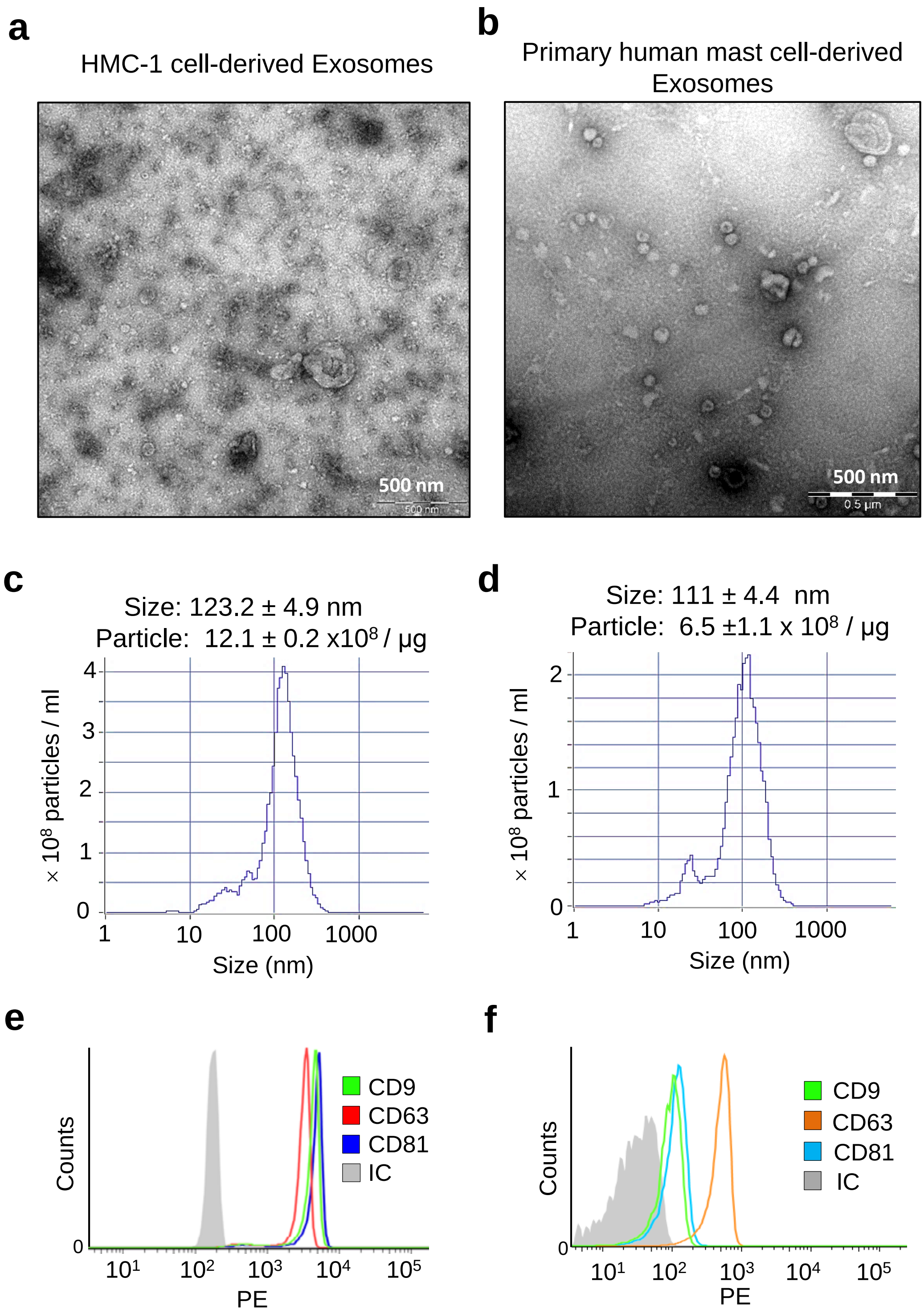

Supplementary Figure 3

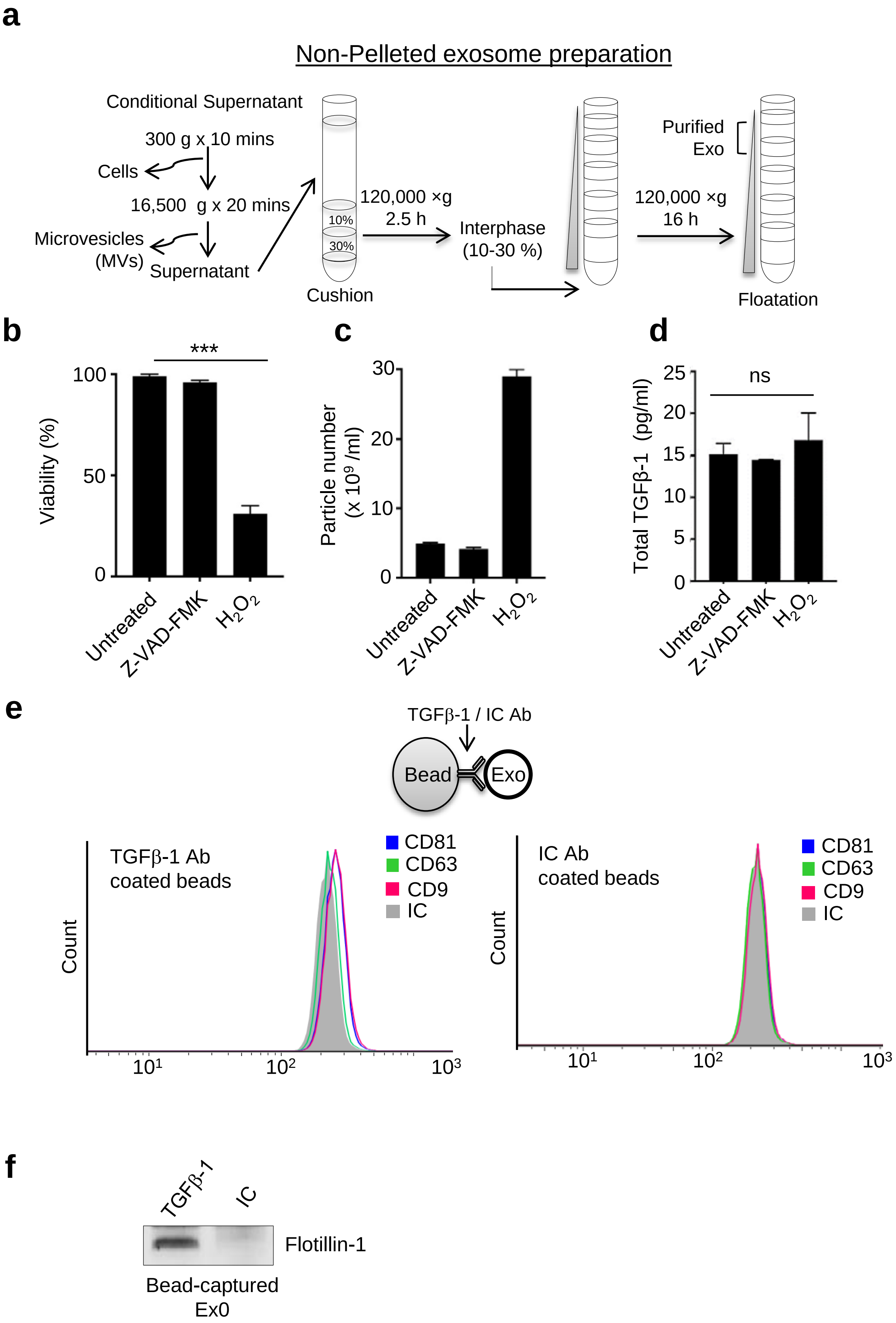

Supplementary Figure 4

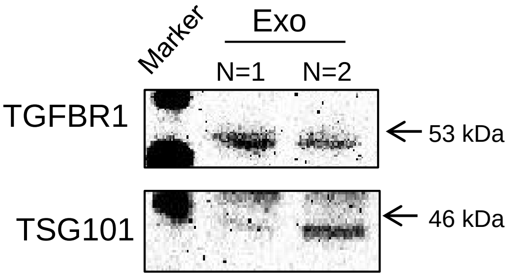

Supplementary Figure 5

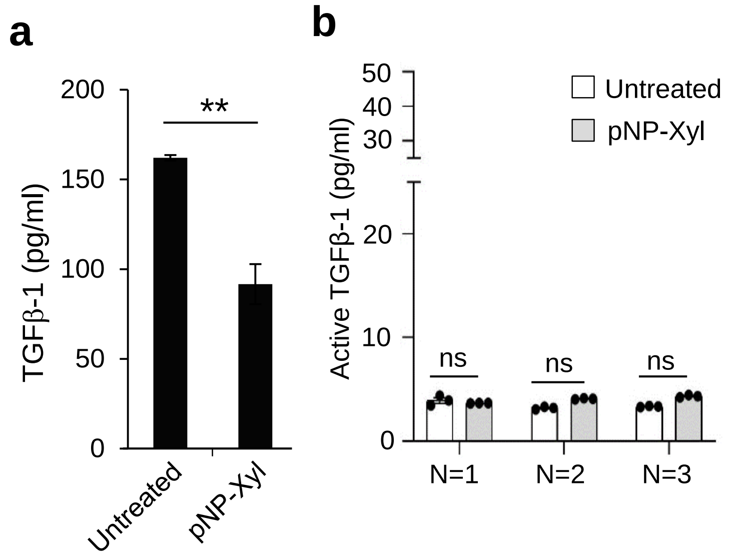

Supplementary Figure 6

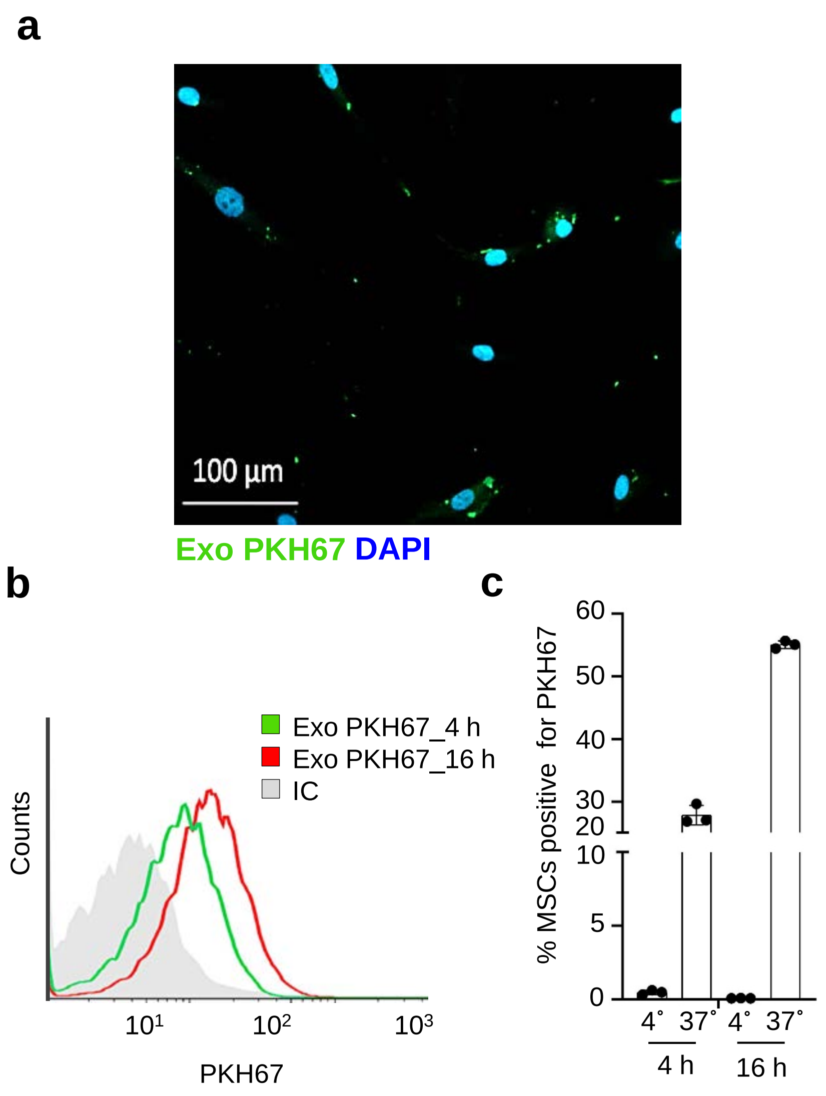

Supplementary Figure 7

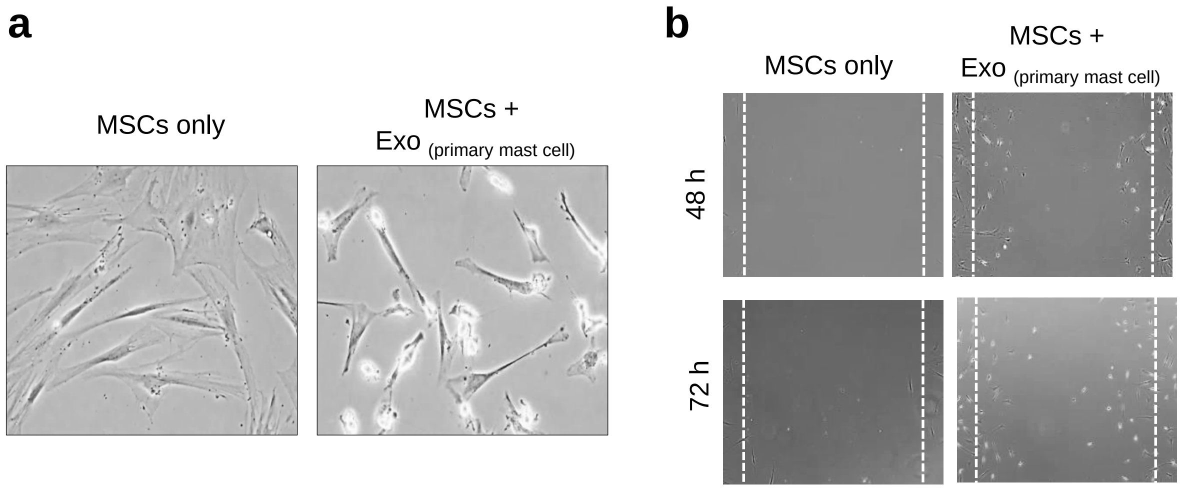

Supplementary Figure 8

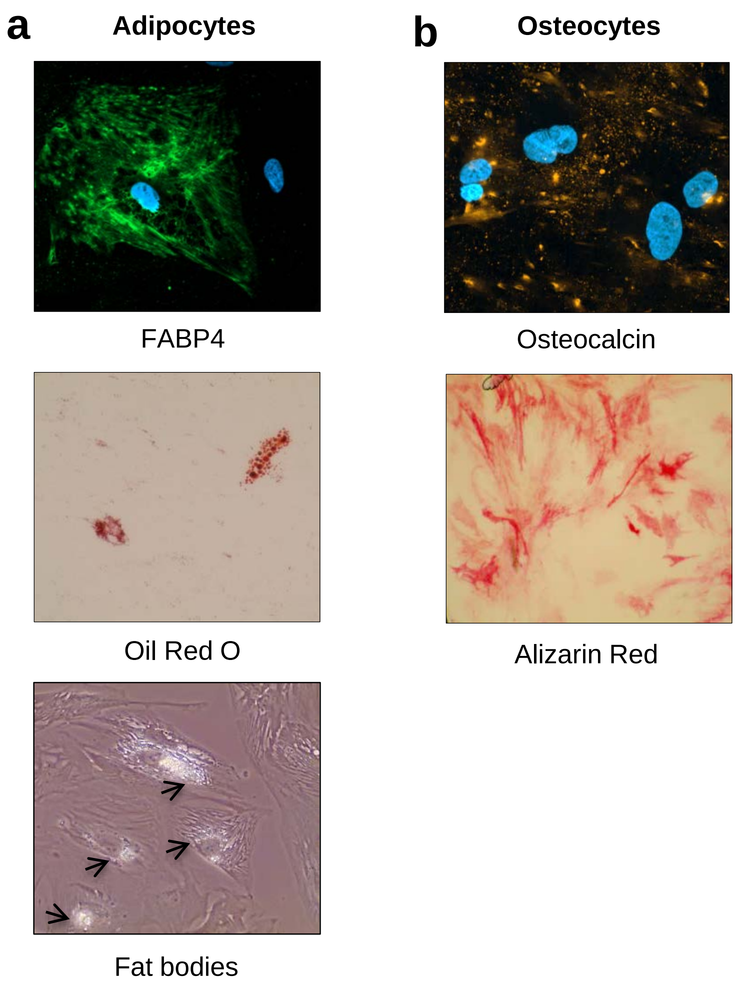

Supplementary Figure 9

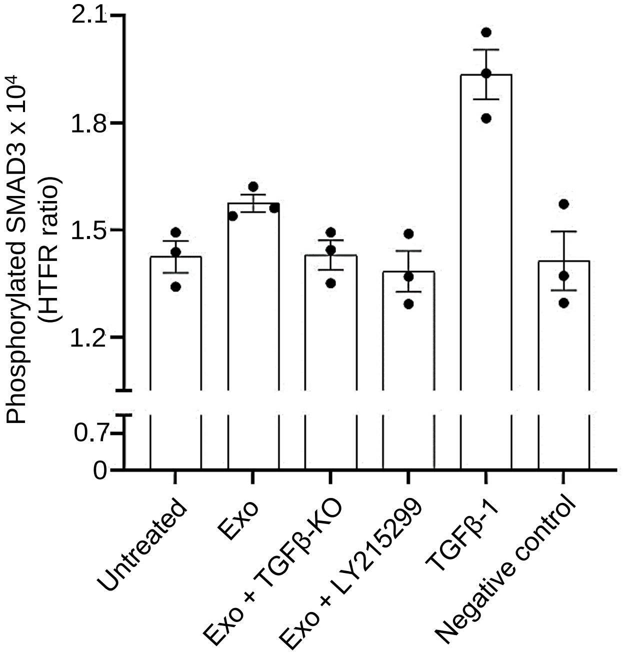

Supplementary Figure 10

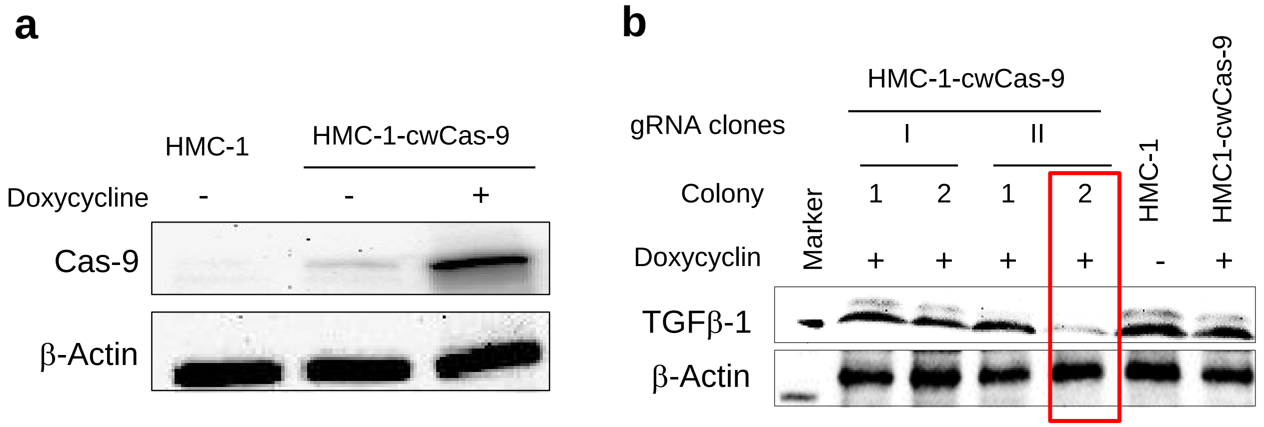

Supplementary Figure 11

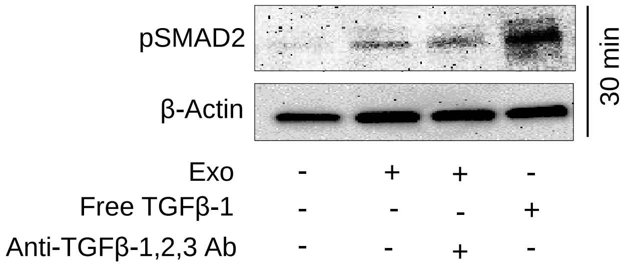

Supplementary Figure 12

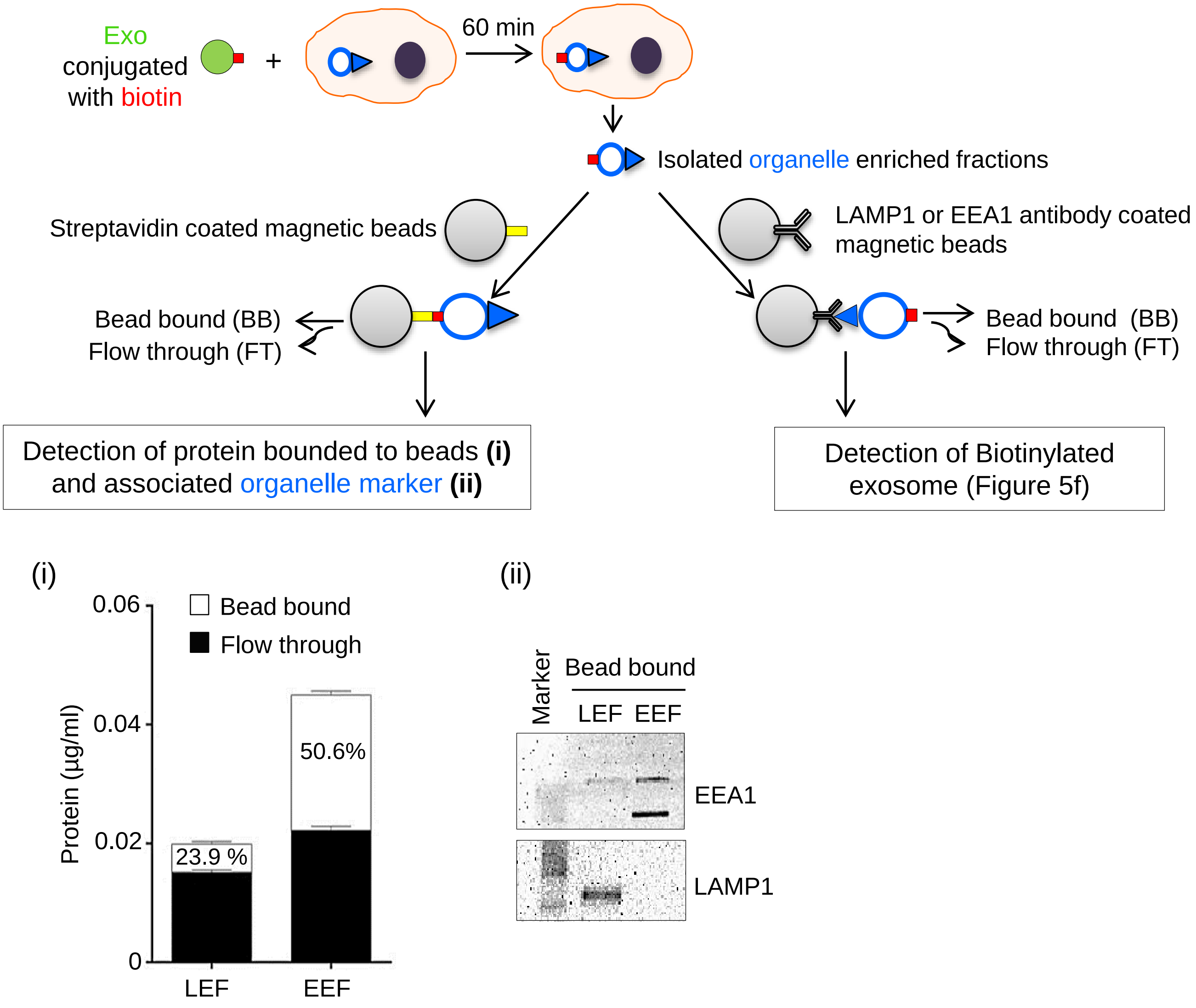

Supplementary Figure 13

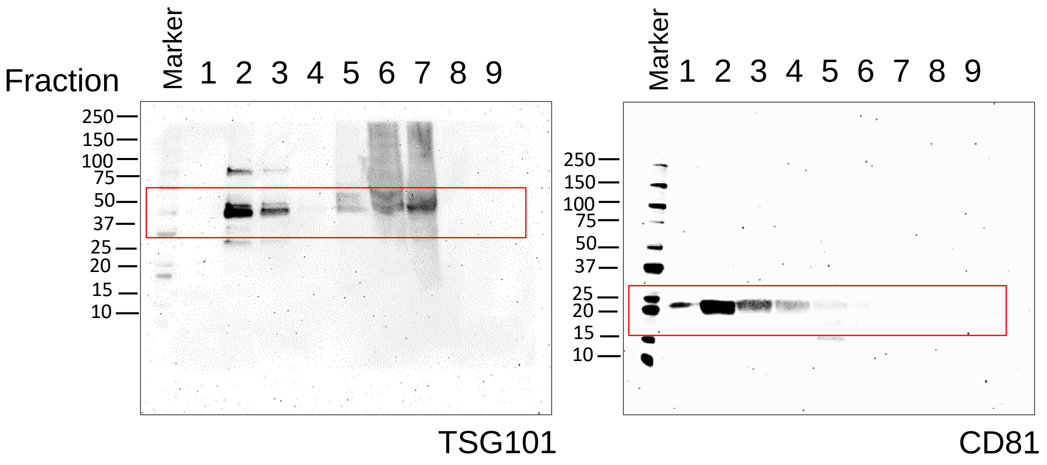

Uncropped image for Figure 1a

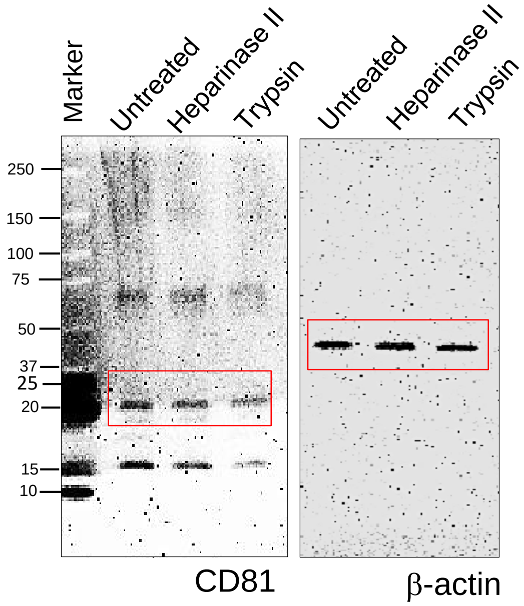

Uncropped image for Figure 2c

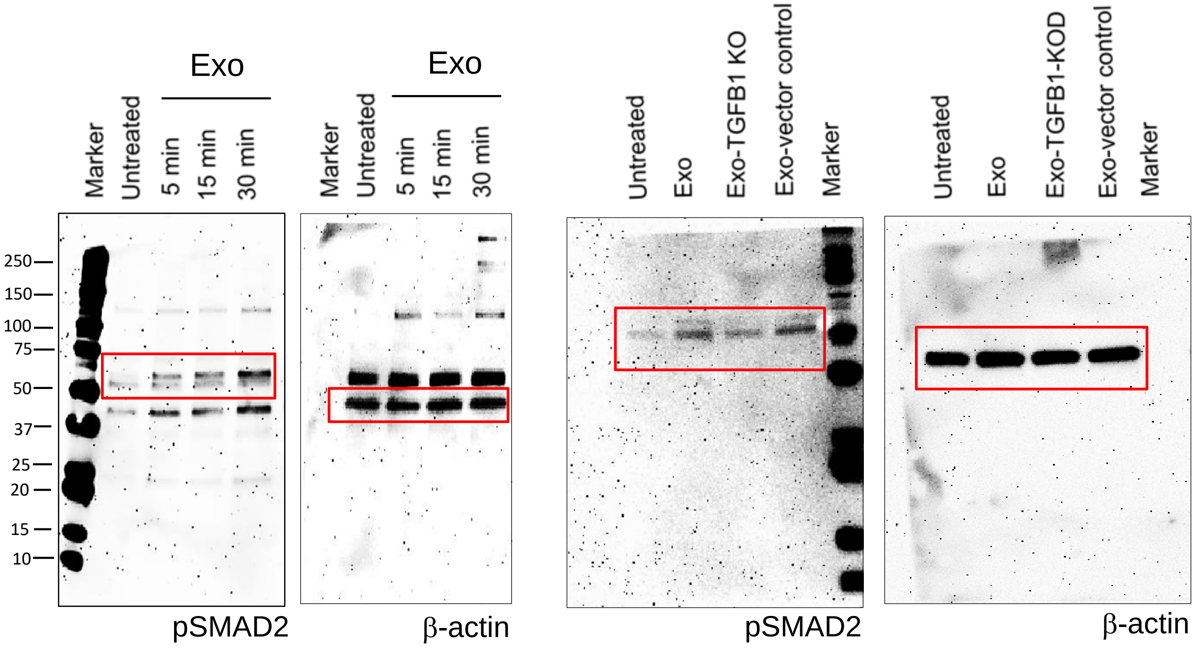

From Figure 4a

From Figure 4e

Uncropped image for Figure 4

Supplementary Figure 14

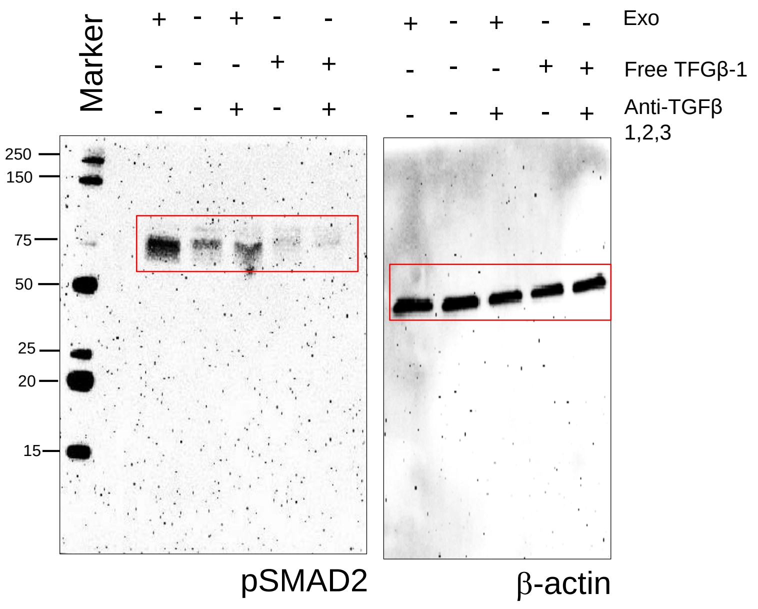

Uncropped image for Figure 5a

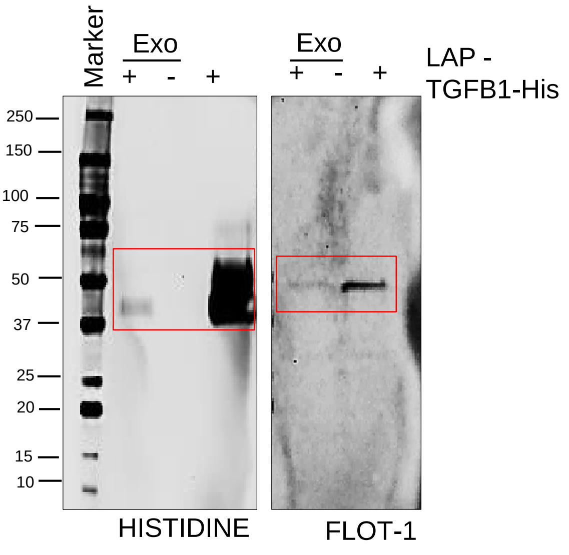

Uncropped image for Figure 6b

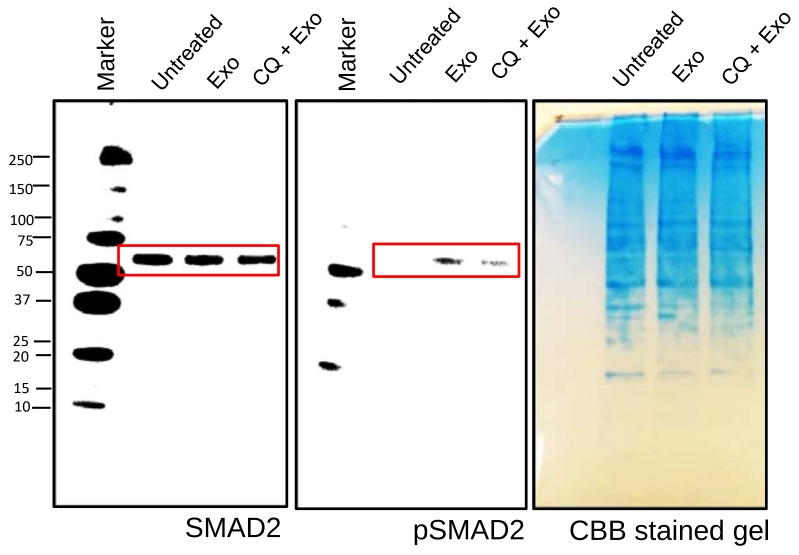

Uncropped images for Figure 6e

Supplementary Figure 15

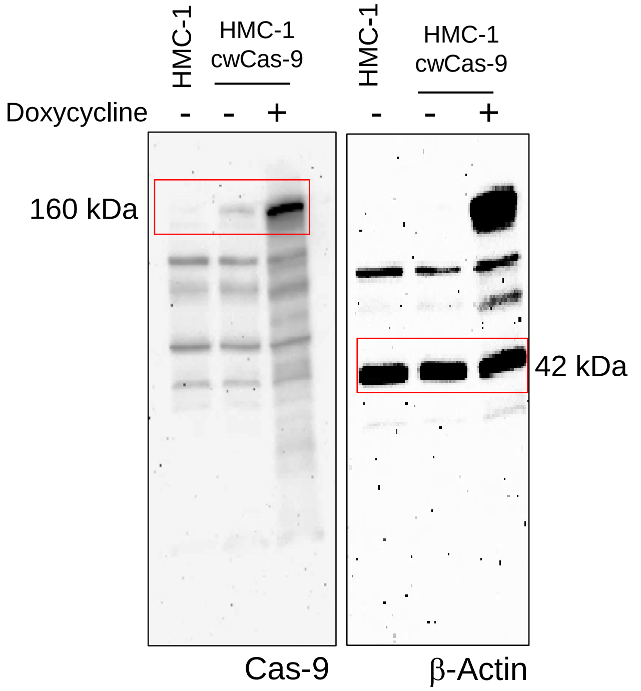

Uncropped image for Supplementary Figure 9a

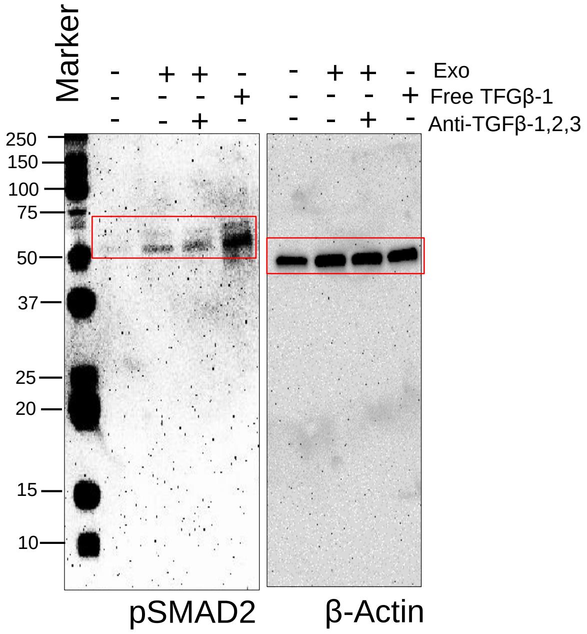

Uncropped image for Supplementary Figure 11

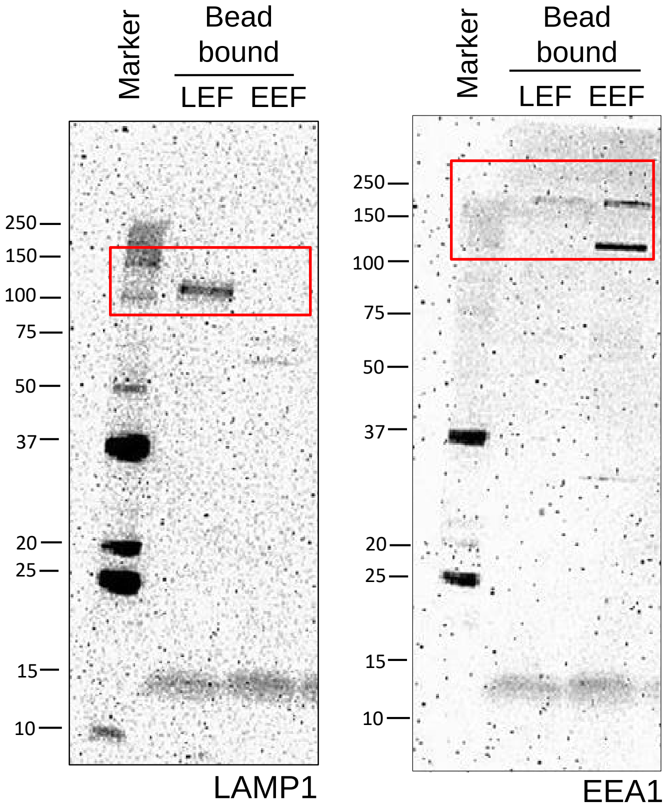

Uncropped images for Supplementary Figure 12 (ii)
