# Supplementary material for: Endosomal signalling via exosome surface TGFβ-1
Source: J Extracell Vesicles. 2019 Sep 20;8(1):1650458. doi: 10.1080/20013078.2019.1650458 (PMC6764367; doi:10.1080/20013078.2019.1650458)
Supplement: Supplemental Material [file ZJEV_A_1650458_SM9071.zip › ZJEV_A_1650458_Supplementary/Supplemental_Data__Figure_legend.pdf]

Supplemental Data

Endosomal signalling via exosome surface TGFβ-1

Ganesh Vilas Shelke [a,b*†], Yanan Yin [a,c*], Su Chul Jang [a], Cecilia Lässer [a], Stefan Wennmalm [d], Hans Jürgen Hoffmann[e,f], Li Li[g], Yong Song Gho[h], Jonas Andreas Nilsson [b] and Jan Lötvall [a]

[a] Krefting Research Centre, Institute of Medicine, the Sahlgrenska Academy, University of Gothenburg, Gothenburg, Sweden
[b] Department of Surgery, Institute of Clinical Sciences, the Sahlgrenska Academy, University of Gothenburg, Gothenburg, Sweden
[c] Department of Biochemistry and Molecular Cell Biology, Shanghai Jiao Tong University, School of Medicine, Shanghai, China
[d] Royal Institute of Technology-KTH, Department of Applied Physics, Experimental Biomolecular Physics Group, SciLife Laboratory, Solna, Sweden
[e] Department of Clinical Medicine, Aarhus University, Aarhus, Denmark
[f] Department of respiratory and Allergy, Aarhus University Hospital, Aarhus, Denmark
[g] Department of Laboratory Medicine, Shanghai First People's Hospital, Shanghai JiaoTong University, Shanghai, China
[h] Department of Life Sciences, Pohang University of Science and Technology, Pohang, Republic of Korea.

[*]These two authors contributed equally to this work.
[†]Current affiliation: Department of Surgery, Institute of Clinical Sciences, the Sahlgrenska Academy, University of Gothenburg, Gothenburg, Sweden.

*Corresponding author: Jan Lötvall,
Krefting Research Centre, University of Gothenburg, Box 424, 405 30 Gothenburg, Sweden
Email: jan.lotvall@gu.se

Legends for supplementary data

Supplementary Table 1. List of proteins obtained from membrane proteomics from mast cell-derived exosomes with the number of peptide hits.

Supplementary Figure 1. TGFβ-1 present in primary mast cell-derived exosomes. Exosomes isolated from primary mast cells were floated on iodixanol density gradients, and the expression of total TGFβ-1 was determined in all fractions using ELISA (n=5, volume 50-100 ml).

Supplementary Figure 2. Characterization of mast cell-derived exosomes. (a, b) The exosomes from HMC-1 **(a)** and primary matured human mast cells **(b)** was evaluated by negative staining and electron microscopy (scale bar = 500 nm). **(c, d)** Representative size distribution and particle concentration of exosomes from primary HMC-1 and matured human mast cells using nanoparticle-tracking analysis (n=3, 300 ml and 50-100 ml respectively). **(e, f)** Exosomes from HMC-1 and primary matured human mast cells were captured on anti-CD63-coated beads, and the presence of CD63, CD81, and CD9 was determined with flow cytometry.

Supplementary Figure 3. Characterization of exosomes obtained by cushion and floatation. (a) Outline of the steps used to obtain exosomes from a density cushion interphase and refloating them on a density gradient to obtain "non-aggregated" purified exosomes. **(b, c)** Viability of HMC-1 cells and particle numbers in purified exosomes obtained after inhibition of internal apoptosis using pan-caspase inhibitor (Z-VAD-FMK) and apoptosis induction (H_2O_2). **(d)** Levels of TGFβ-1 on these exosomes released from HMC-1 after apoptosis inhibition and induction. **(e, f)** Exosomes obtained from the above method were captured on TGFβ-1 or isotype (IC) antibody-coated beads and were probed for the presence of exosomes enriched surface proteins (CD63, CD81, and CD9) and the luminal marker flotilllin-1 with flow cytometry and immunoblotting, respectively.

Supplementary Figure 4. Mast cell-derived exosomes harbor TGFβ receptor-1. The presence of TGFBR1 and TSG101 in exosomes using immunoblotting using (n=2. Volume =600 ml).

Supplementary Figure 5. pNP-Xyl dependent association of exosomes with total TGFβ-1 and active TGFβ-1. (a) Estimation of total TGFβ-1 and (**b**) active TGFβ-1 using ELISA. (n=3, volume 600 ml and n=3, 200-600 ml respectively).

Supplementary Figure 6. Exosomes uptake by MSCs is an active process. (a) Immuno-fluorescence image of MSCs that had been taking up PKH67-labeled exosomes after 4 hours of co-incubation (scale bar=100 μm). (**b**) Flow cytometry analysis of MSCs that were incubated with PKH67-labeled exosomes for 4 h or 16 h. **(c)** Percentage of MSCs that were positive for PKH67-labeled exosomes after incubation at 4°C or 37°C for 4 h or 16 h.

Supplementary Figure 7. Primary human mast cell-derived exosomes enhance the migration of primary human mesenchymal stem cells. **(a)** Morphology of MSCs incubated with exosomes derived from primary human mast cells and visualized using light microscopy. **(b)** An *in-vitro* wound healing scratch assay was performed on monolayers of untreated or exosome-treated MSCs (n = 2) from primary human mast cells. MSCs were imaged at 48 h and 72 h after injury.

Supplementary Figure 8. Exosomes do not alter the multipotency of MSCs. MSCs were incubated with mast cell-derived exosomes for 48 h and then differentiated into the adipocytic and osteocytic phenotypes. After 15 days of culturing MSCs in corresponding differentiation medium, the cells were labeled with lineage-specific markers for **(a)** adipocytes (FABP4, Oil Red O and fat bodies), and **(b)** osteocytes (Osteocalcin and Alizarin Red).

Supplementary Figure 9. Exosomes-associated TGFβ-1 activates phosphorylation of SMAD3. Level of phosphorylated SMAD3 was detected in with the FRET assay in MSC cell lysate after treatment with mast cell-derived exosomes for 30 min.

Supplementary Figure 10. Doxycycline-regulated Cas9-expression system in mast cells (a) Immunoblotting analysis of FLAG-tagged Cas-9 nuclease, regulated by doxycycline, in HMC-1 cells transduced with pCW-Cas9. **(b)** The efficiency of TGFB-1 gRNA clones in Cas-9-expressing HMC-1 cells was estimated by immunoblotting of TGFβ-1 in various clones.

Supplementary Figure 11. Exosome-associated TGFβ-1 regulates activation of TGFβ-1 signaling. Immunoblot analysis of pSMAD2 in MSCs after 30 minutes treatment with free TGFβ-1 or exosomes, as well as after blocking the exosome-associated TGFβ-1.

Supplementary Figure 12. Outline of the method to isolate the lysosome-enriched and endosome-enriched compartments. Isolated organelle fractions were incubated independently with streptavidin-coated magnetic beads (left part) or organelle-specific (LAMP1 or EEA1) antibody-coated magnetic beads

(right side). **(i)** Total protein in the bead-bound and flow-through fractions was measured to evaluate the biotinylated protein in the LEF and EEF. **(ii)** Organelle-specific markers (LAMP1 or EEA1) were evaluated by immunoblotting to detect their presence in the LEF and the EEF that had bound to the streptavidin-coated beads. In parallel (right side), the presence of biotinylated proteins in organelles isolated from magnetic beads coated with LAMP1 or EEA1 antibodies was evaluated by immunoblotting (Fig. 5f) with a streptavidin antibody.

Supplementary Figure 13. Uncropped western blot images for figure 1a, 2c, 4a and 4e.

Supplementary Figure 14. Uncropped western blot images for supplementary figure 5a, 6b and 6e.

Supplementary Figure 15. Uncropped western blot images for supplementary Figure 9a, 11 and 12 (ii).
